# Narcolepsy in Parkinson's disease with insulin resistance

**DOI:** 10.12688/f1000research.27413.1

**Published:** 2020-11-23

**Authors:** Alisha Chunduri, Wim E. Crusio, Anna Delprato

**Affiliations:** 1 Department of Biotechnology, Chaitanya Bharathi Institute of Technology, Hyderabad, 500075, India; 2 Department of Research and Education, BioScience Project, Wakefield, MA, 01880, USA; 3 Institut de Neurosciences Cognitives et Intégratives d'Aquitaine, CNRS UMR 5287, Pessac, 33615, France; 4 Institut de Neurosciences Cognitives et Intégratives d'Aquitaine, UMR 5287 University of Bordeaux, Pessac, 33615, France

**Keywords:** Parkinson’s disease, narcolepsy, insulin resistance, diabetes, circadian

## Abstract

**Background: **Parkinson’s disease (PD) is characterized by its progression of motor-related symptoms such as tremors, rigidity, slowness of movement, and difficulty with walking and balance. Comorbid conditions in PD individuals include insulin resistance (IR) and narcolepsy-like sleep patterns. The intersecting sleep symptoms of both conditions include excessive daytime sleepiness, hallucinations, insomnia, and falling into REM sleep more quickly than an average person. Understanding of the biological basis and relationship of these comorbid disorders with PD may help with early detection and intervention strategies to improve quality of life.

**Methods: **In this study, an integrative genomics and systems biology approach was used to analyze gene expression patterns associated with PD, IR, and narcolepsy in order to identify genes and pathways that may shed light on how these disorders are interrelated. A correlation analysis with known genes associated with these disorders *(LRRK2, HLA-DQB1, and HCRT*) was used to query microarray data corresponding to brain regions known to be involved in PD and narcolepsy. This includes the hypothalamus, dorsal thalamus, pons, and subcoeruleus nucleus. Risk factor genes for PD, IR, and narcolepsy were also incorporated into the analysis.

**Results: **The PD and narcolepsy signaling networks are connected through insulin and immune system pathways. Important genes and pathways that link PD, narcolepsy, and IR are *CACNA1C, CAMK1D, BHLHE41, HMGB1,* and AGE-RAGE.

**Conclusions: **We have identified the genetic signatures that link PD with its comorbid disorders, narcolepsy and insulin resistance, from the convergence and intersection of dopaminergic, insulin, and immune system related signaling pathways. These findings may aid in the design of early intervention strategies and treatment regimes for non-motor symptoms in PD patients as well as individuals with diabetes and narcolepsy.

## Introduction

Parkinson’s disease (PD) is characterized by its progression of motor-related symptoms such as tremors, rigidity, slowness of movement, and difficulty with walking and balance ^1^. The motor difficulties associated with PD are attributed to the loss of dopaminergic neurons in the substantia nigra ^1^. There are also non-dopamine lesions that are involved in PD that include the caudal group of intralaminar nuclei (located in dorsal thalamus), and subcoeruleus nuclei ^1^. The main genetic cause of PD is attributed to mutation of the *LRRK2* (leucine-rich repeat kinase 2) gene ^2, 3^.

Sleep disruption is another manifestation of *LRRK2*-PD and is often more disturbing than the motor symptoms ^4^. Most PD patients have daytime sleep attacks and REM sleep disorder that resemble narcolepsy associated sleep symptoms such as excessive daytime drowsiness, sleep paralysis, hallucinations ^5^, and in some cases episodes of cataplexy ^6^. People with narcolepsy frequently enter REM sleep rapidly, within 15 minutes of falling asleep and the muscle weakness or dream activity of REM sleep can occur during wakefulness or be absent during sleep ^7^. Alleles of the *HLA-DQB1* (major histocompatibility complex, class II, DQ beta 1) gene are associated with a predisposition to narcolepsy ^8^, PD ^9^, and Type I diabetes ^10^. There is also a significantly increased risk of PD among patients with a history of diabetes ^11^.

Besides *HLA-DQB1*, the relationship between PD, narcolepsy, and IR may be in part attributed to the hypocretins/orexins which are produced by the *HCRT* gene ^12^. Hypocretins are neurotransmitters that are manufactured by a small number of neurons in the hypothalamus ^13^. They act to stimulate target neurons and promote wakefulness while suppressing rapid-eye-movement (REM) sleep ^14^. Research has shown that there is a massive loss of hypocretin neurons in patients of both PD and narcolepsy and it is hypothesized that the reduction of hypocretin may be the underlying pathogenesis of the narcoleptic symptoms in PD ^5, 15, 16^. In addition to their role in narcolepsy and PD, hypocretins modulate glucose and insulin metabolism ^15^. In this study we explore the connection between PD, narcolepsy, and IR using an integrative genomics and systems biology approach.

## Methods

### Genesets and evaluation

Microarray data was collected from the Allen Brain Database using the Human Brain Atlas. To obtain the data, a gene search for *LRRK2, HLA-DQB1*, and *HCRT* was performed. Each of these genes were used to query the atlas for correlates to the hypothalamus, dorsal thalamus, pons, and subcoeruleus nucleus using the dropdown menu for each of the six donor post-mortem brains available in the Allen Human Brain Atlas.

Genes whose expression pattern correlated with *LRRK2, HLA-DQB1*, and *HCRT* were collected for analysis. Correlates with a range of Pearson r values from 0.6 to 1.0 were considered in the analysis ( *Extended data*, Workbook 1 ^17^). The rationale was to investigate genes with a similar expression pattern in order to identify gene correlates specific and common to *LRRK2, HLA-DQB1*, and *HCRT*. Risk factor genes and genes contributing to PD, narcolepsy, and IR were obtained from OMIM, Harmonizome, and GeneWeaver.

Each geneset was evaluated using Gene Ontology (GO) enrichment for clustering, pathways, and keywords using the Database for Annotation, Visualization and Integrated Discovery (DAVID, version 6.8) and the Gene Ontology databases with integrated tools for analysis. Clustering was done in DAVID using the default parameters which include medium stringency settings and a kappa similarity value of 3. The Benjamini corrected P-value was used to determine enrichment significance. The pathway enrichment was performed using KEGG and Panther pathways. The pathways were analyzed manually and evaluated based on shared themes. For the keyword enrichment, a keyword search of the DAVID functional annotation table output was used to identify genes associated with relevant traits related to *LRRK2, HLA-DQB1*, and *HCRT* function. The keywords considered were ‘sleep’, ‘circadian’, ‘parkinson’, ‘locomotion’, ‘dopamine’, ‘behavior’, ‘learning’, ‘memory’, and ‘transcription factor’. Geneset overlap was assessed using Venny 2.0, an online program that compares lists of items to determine the common and unique genes between *LRRK2, HLA-DQB1*, and *HCRT* within and among each brain region (hypothalamus, dorsal thalamus, pons, and subcoeruleus nucleus). Geneset overlap was visualized with the UpSet Library in RStudio, R Version 4.0.2.

### Network analysis

The String database (version 11.0) was used to build a protein-protein interaction network (PPI) for LRRK2, HLA-DQB1, HCRT and CAMK1D which was identified in this study as the only common risk factor gene associated with PD, narcolepsy and IR (Results section: "Functional analysis of PD, narcolepsy and IR risk factor and related genes"). The network was constructed based on experimentally validated interactions using the medium confidence score of 0.4. The combined scores for the interactions are computed by combining the probabilities from the different evidence channels and corrected for the probability of randomly observing an interaction. First and 2nd shell interactions are included in the network. The network was exported from STRING and analyzed in Cytoscape (version 3.7). Network bottlenecks and clusters were identified with Cytoscape plugins CytoHubba (version 0.1) and MCODE (version 1.6.1), respectively. ClueGo (version 2.5.7) was used to analyze the common risk factors and contributing genes for PD, narcolepsy, and IR. The nodes in the network have been manually arranged for proper visibility. Select enriched terms are included in the network ( Figure 3A). All of the enriched terms are provided in *Extended data*, Workbook 5, sheet 5 ^18^.

## Results

### Functional analysis of gene correlates

The cluster analysis for the *LRRK2, HLA-DQB1*, and *HCRT* gene correlates for each brain region resulted in significant enrichment categories for only *HLA-DQB1* related genesets. For *LRRK2* and *HCRT* there are several instances in which clusters contained enrichment terms for insulin, diabetes, PD, other neurodegenerative disorders, and circadian processes but these did not achieve significance based on the corrected P value criteria. Also, of note for almost every set of correlates, there were many significant enrichment categories and corresponding genes associated with keratinocytes/keratin and olfaction. The clustering results for each set of gene correlates are listed in *Extended data*, Workbook 2 ^19^.

For the *HLA-DQB1* clusters, the significant enrichment terms are: dorsal thalamus: hsa05012:Parkinson's disease (P=4.38E-06), 31 genes and hsa04940:Type I diabetes mellitus (P=0.03), 10 genes; subcoeruleus nucleus: hsa04940:Type I diabetes mellitus (P=6.13E-05), 15 genes, and pons: hsa04940:Type I diabetes mellitus, six genes (P=0.001). The other genes and enrichment categories clustering with PD in the dorsal thalamus are related to mitochondria processes such as oxidative phosphorylation and electron transport as well as Alzheimer’s disease (AD) and Huntington disease (HD).

### Geneset overlap

**Figure 1.**
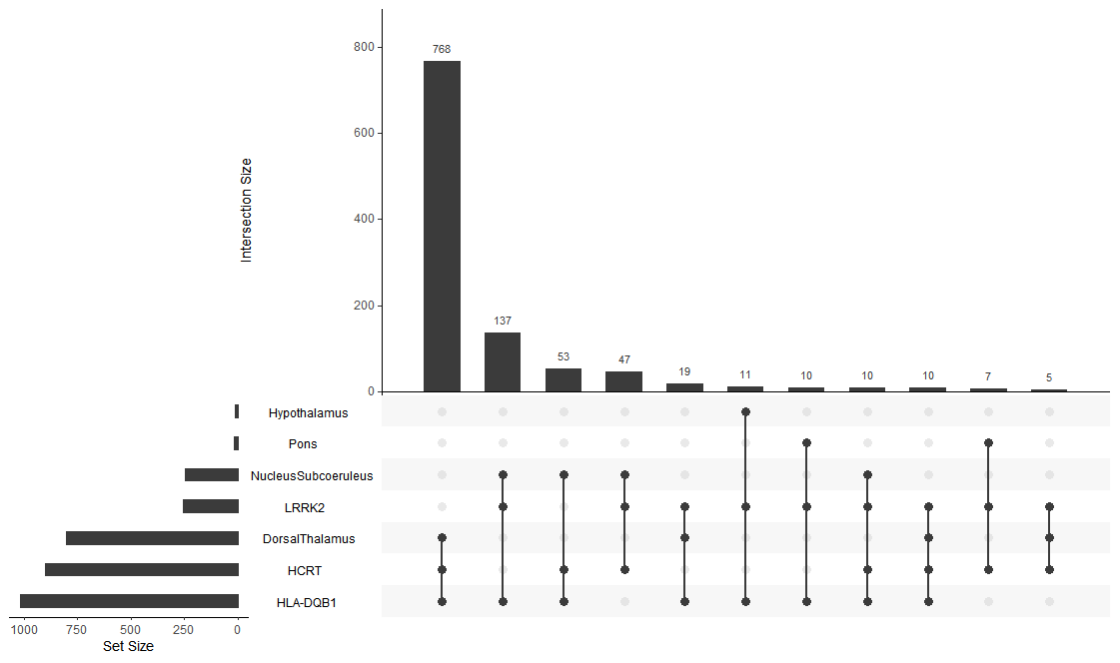
Geneset overlap. Shared correlates for LRRK2, HLA-DQB1 and HCRT over all brain regions. X-axis, Intersection size; Y-axis, Gene number and brain regions.

Among the sets of gene correlates for *LRRK2, HLA-DQB1* and *HCRT*, there are 10 common genes in both the dorsal thalamus and subcoeruleus nucleus ( *HNRNPU, GDF11, ROCK1, GABRA4, PFKFB2, ZNF846, PAK2, A_32_P232747, SLC9A3R2,* and *DISC1*; Figure 1B). Among the relevant genes are *ROCK1,* which is involved in negative regulation of neuron apoptotic processes, *DISC1* associated with neuron migration, and *HNRNPU* involved in circadian regulation of gene expression. The ten common genes in the subcoeruleus nucleus are *LAMP2, LOC653110, HNRNPU, SLC6A6, PHTF2, ITGB2, OPA3, GLYAT, HCN4,* and *HMGB1,* among which the relevant ones to this study are *HNRNPU,* which as mentioned above is involved in circadian regulation of gene expression and insulin signaling, *ITGB2*, which is associated with PD and IR, and *HMGB1,* which is a ligand for the RAGE receptor. Among the sets of gene correlates for *LRRK2, HLA-DQB1*, and *HCRT*, there are no common correlated genes in the hypothalamus and pons.

A detailed description of all shared genes and their associated function for each brain region is provided in *Extended data*, Workbook 3 (sheets 1–8) ^20^. Briefly, the dorsal thalamus and subcoeruleus nucleus have the largest number of shared correlates between *LRRK2, HCRT*, and *HLA-DQB1*. Many of these genes for both brain regions are associated with neuron, insulin, and dopamine related processes. There are also several genes connected directly to PD. In sharp contrast, however, the dorsal thalamus associated correlates have many genes linked to circadian function.

In the dorsal thalamus, the relevant genes are associated with neuron function (negative regulation of neuron apoptotic process, neuron projection development and regulation, neuron differentiation, neuron fate commitment, neuron death in response to oxidative stress, neuron regeneration), circadian processes (regulation of circadian rhythm, circadian entrainment, circadian regulation of gene expression, regulation of circadian rhythm, entrainment of circadian clock by photoperiod), and insulin signaling (insulin secretion, insulin receptor signaling, insulin secretion). Other genes of interest are related to dopamine (dopaminergic neuron differentiation, regulation of dopamine uptake involved in synaptic transmission, positive regulation of dopamine secretion, Wnt signaling pathway involved in midbrain dopaminergic neuron differentiation, dopamine receptor binding, dopamine biosynthetic process, adenylate cyclase-activating dopamine receptor signaling pathway) and also behavior (locomotory, feeding, learning, memory and vocalization, response to stimulants).

In the subcoeruleus nucleus, the relevant genes are also associated with neuron function (negative regulation of neuron differentiation, dopaminergic neuron differentiation, neuron apoptotic process, negative regulation of neuron differentiation, forebrain neuron differentiation), insulin signaling (insulin secretion, insulin receptor signaling pathway, negative regulation of insulin receptor signaling pathway, positive regulation of insulin secretion, diabetes mellitus), dopamine related processes (dopaminergic synapse, dopamine biosynthetic process, dopaminergic neuron differentiation, regulation of synaptic transmission, dopaminergic dopamine biosynthetic process from tyrosine), and behavior (locomotory, vocal learning, response to fear, grooming, response to stimulants).

There are few shared correlated genes in the hypothalamus and pons. For the hypothalamus, the most pertinent genes are involved in neuron migration and circadian processes. In the pons, the relevant genes are concerned with negative regulation of neuron apoptotic processes, neuron projection, circadian regulation of gene expression, and hippocampus and pyramidal neuron development.

Geneset overlap of the correlates for *LRRK2, HLA-DQB1,* and *HCRT* was assessed within each brain region (see *Extended data*, Workbook 3, sheets 9–11 ^20^). Of the *LRRK2* correlates,1.6% were common in all 4 brain regions. Among these are genes associated with insulin ( *MAX, NUCKS1, PIK3R1, PTPN11*), diabetes ( *PIK3R1*) and circadian-related processes ( *HNRNPU, BHLHE41*). Several transcription factors were also present ( *MAX, SKI, ATF7IP, NUCKS1, BHLHE41, NR2C2, PIK3R1*). One of these, *BHLHE41*, acts as a negative regulator of orexin, controls circadian rhythms, and is associated with short sleep syndrome and advanced sleep phase disorder. For *HLA-DQB1*, 1.2% of the correlates are common in all brain regions; relevant associated themes include PD and dopamine ( *SLC18A1*) insulin ( *HLA-DRB5, HLA-DOA, HLA-DQA1, HLA-DQB1*) and transcription factors ( *PYCARD, SOX8, FOXE3, LGALS9, HMGB1, ZNF446*). Only 0.2% of the *HCRT* correlates are common among all brain regions considered. This includes *HCRT* itself and an insulin associated gene, *GHSR*. Of note, the *MOG* gene, which is present among the *HLA-DQB1* correlates of the subcoeruleus nucleus and *HCRT* correlates of the hypothalamus, is a risk factor for narcolepsy and is linked to PD.

### Keyword evaluation of gene correlates

**Figure 2.**
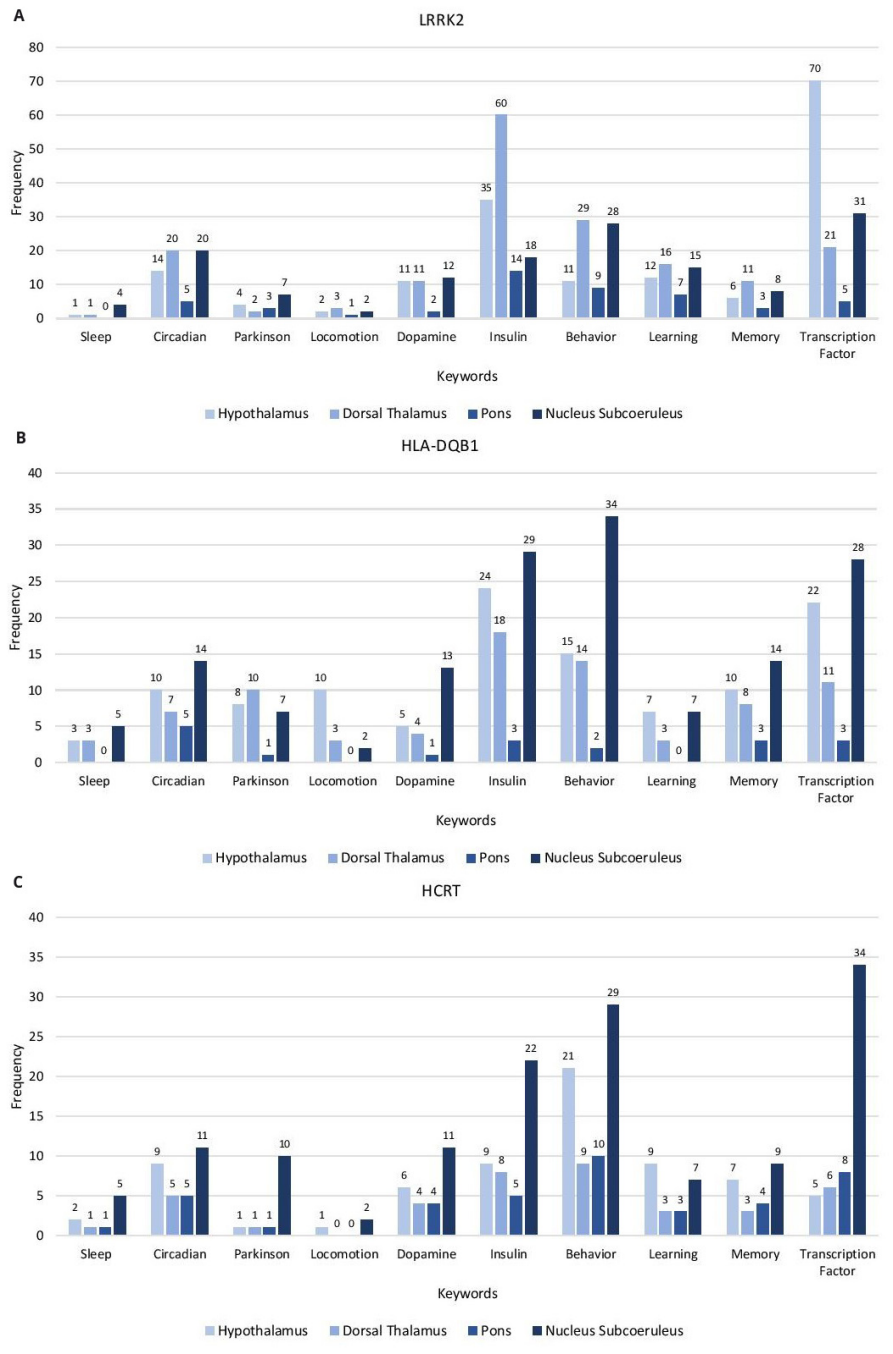
Keyword enrichment. Representative keyword enrichment of the gene correlates of LRRK2, HLA-DQB1 and HCRT in the Hypothalamus, Dorsal Thalamus, Pons and Nucleus Subcoeruleus based on GO term classification. ( **A**) LRRK2 gene correlates ( **B**) HLA-DQB1 gene correlates ( **C**) HCRT gene correlates. X-axis, keyword categories; Y-axis, frequency of occurrence.

From the GO analysis of the gene correlates, a functional annotation table was generated for GO Biological Process. Genes associated with keywords were obtained and their frequencies determined. The keyword categories used are as follows: sleep, circadian, Parkinson, locomotion, dopamine, insulin, behavior, learning, memory, and transcription factor ( Figure 2A–C and *Extended data*, Workbook 4, sheets 1–6 ^21^). Each of the correlates for the genesets are evaluated for keywords related to the phenotypes of narcolepsy, PD, and IR in the hypothalamus, dorsal thalamus, pons, and subcoeruleus nucleus. Most of the keywords of the three sets of gene correlates are associated with subcoeruleus nucleus.

The *LRRK2* gene correlates have the highest frequency of the keyword categories. The highest represented categories are: transcription factor (hypothalamus), insulin, behavior, learning, memory, locomotion (dorsal thalamus), dopamine, Parkinson, and sleep (subcoeruleus nucleus) and circadian processes (equal frequency in dorsal thalamus and subcoeruleus nucleus).

The highest represented keyword categories for *HLA-DQB1* are behavior, insulin, transcription factor, circadian, memory, dopamine, sleep (subcoeruleus nucleus), locomotion (hypothalamus), and learning (equal frequencies in the hypothalamus and subcoeruleus nucleus). The highest represented categories of *HCRT* are transcription factor, behavior, insulin, circadian, dopamine, Parkinson, memory, sleep, locomotion (subcoeruleus nucleus), and learning (hypothalamus).

### Functional analysis of PD, narcolepsy, and IR risk factor and related genes

PD, narcolepsy, and IR risk factor and related genesets were evaluated to identify a common set of genes associated with the three disorders ( *Extended data*, Workbook 5, sheets 1–4 ^18^). There were 38 shared genes between the PD and narcolepsy genesets. *CAMK1D* is the only gene common among the three genesets for PD, narcolepsy, and IR and it is a Calcium/Calmodulin kinase that is upregulated in PD patients and is also a risk factor for Type 2 diabetes ^22, 23^.

Of the common PD and narcolepsy genes, several were directly associated with PD and narcolepsy behavioral phenotypes such as locomotion ( *DRD2, DRD3, DRD4, GDNF, SLC18A2*), sleep ( *HTR2A, DRD2, DRD3, GRIN2A, NLGN1*), circadian processes ( *PPARGC1A, CACNA1C, DRD2, DRD3, DRD4, GRIN2, MAPK1, NLGN1*), circadian entrainment ( *CACNA1C, GRIN2A, MAPK1*), learning ( *COMT, DRD1, DRD2, DRD3 GRIN2A*) and memory ( *GRIN2A, HTR2A, COMT, DRD1, DRD2, DRD3*). There were two common genes between the PD and IR genesets: *RREB1,* which is a transcription factor, and *ANKFY1,* which is involved in vesicle trafficking and is also implicated in Type 2 diabetes. There is one common gene between narcolepsy and IR: *HLA-DQB1*, which is the narcolepsy associated gene under study here.

**Figure 3.**
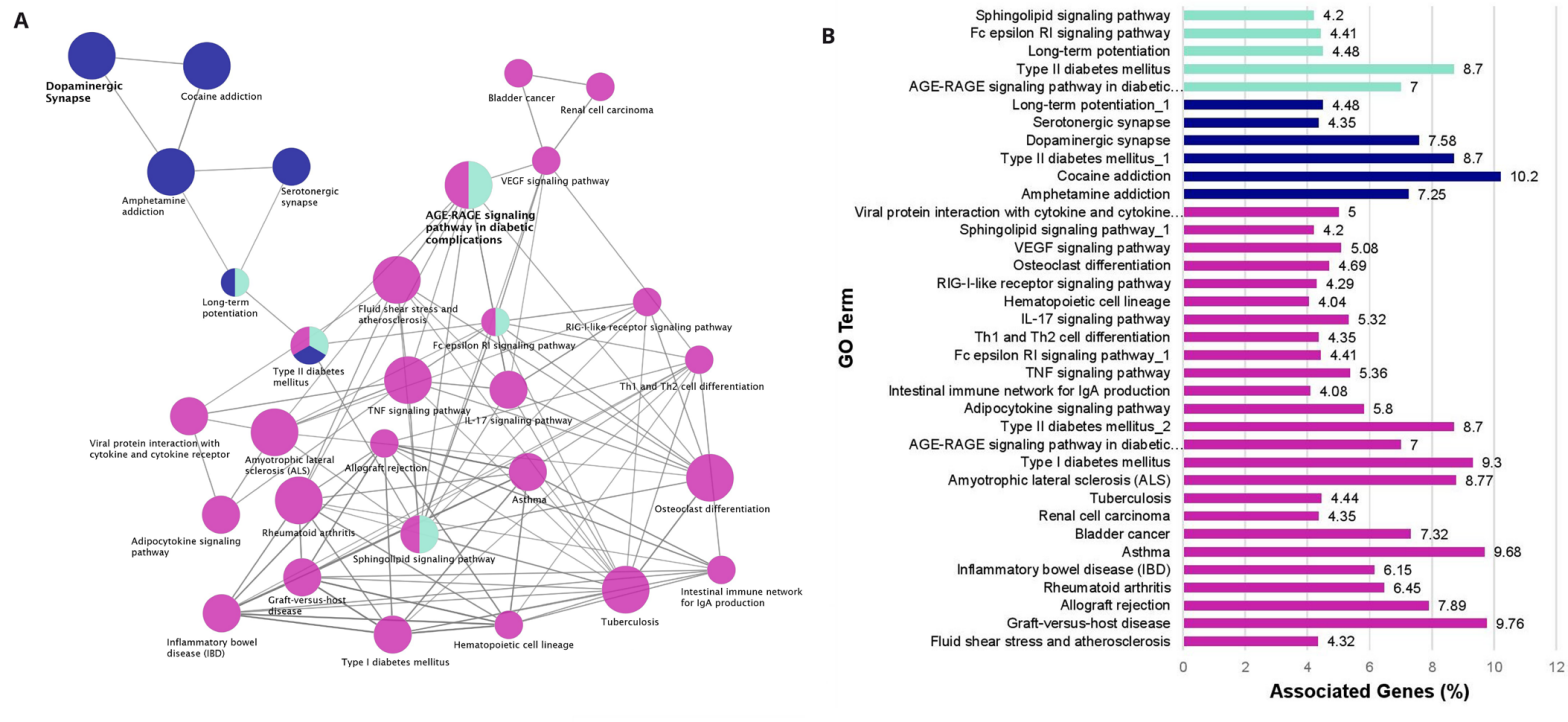
Enrichment network analysis. ( **A**) Risk factors enrichment network. In the network the color gradient indicates the proportion of genes in each cluster associated with the enriched GO term. Dark blue nodes include dopaminergic synapse and pathways related to the reward system. Cyan nodes include the AGE-RAGE Signaling pathway in diabetic complications, immune system pathways and lipid signaling. Magenta nodes involve terms associated with immune system function and also insulin signaling. ( **B**) GO pathway terms and associated genes. Bar graph showing the percentage of genes connected with the GO terms. Bars are colored according to the network ( Figure 3A).

The enrichment results are visualized as a network of functionally grouped terms and pathways and listed in the accompanying bar graph ( Figure 3A, B, *Extended data*, Workbook 5, sheet 5 ^18^). The most significant term of a given group is highlighted as the leading term in the network which is indicated by color. The most significant terms emphasized in the graph are dopaminergic synapse (ten genes, KEGG ID:04728, P=2.77E-09) and the AGE-RAGE signaling pathway in diabetic complications (seven genes, KEGG ID:04933, P=4.70E-06) both of which are relevant to PD and IR. Other relevant enriched GO Terms include Type I diabetes mellitus (four genes, KEGG:04940, P=6.27E-04), Type II diabetes mellitus (four genes, KEGG:04930, P=7.54E-04), and Amyotrophic lateral sclerosis (five genes, KEGG:05014, P=9.56E-05). The other enriched terms in the network also represent pathways linked to the reward system, serotonin signaling, immune system function, and insulin regulation. There are several points of convergence in the graph where the enriched terms overlap: AGE-RAGE, Sphingolipid, and Fc Epsilon RI signaling pathways as well as long term potentiation ( *Extended data*, Workbook 5, sheet 5 ^18^).

### The PD, narcolepsy, and IR connection

**Figure 4.**
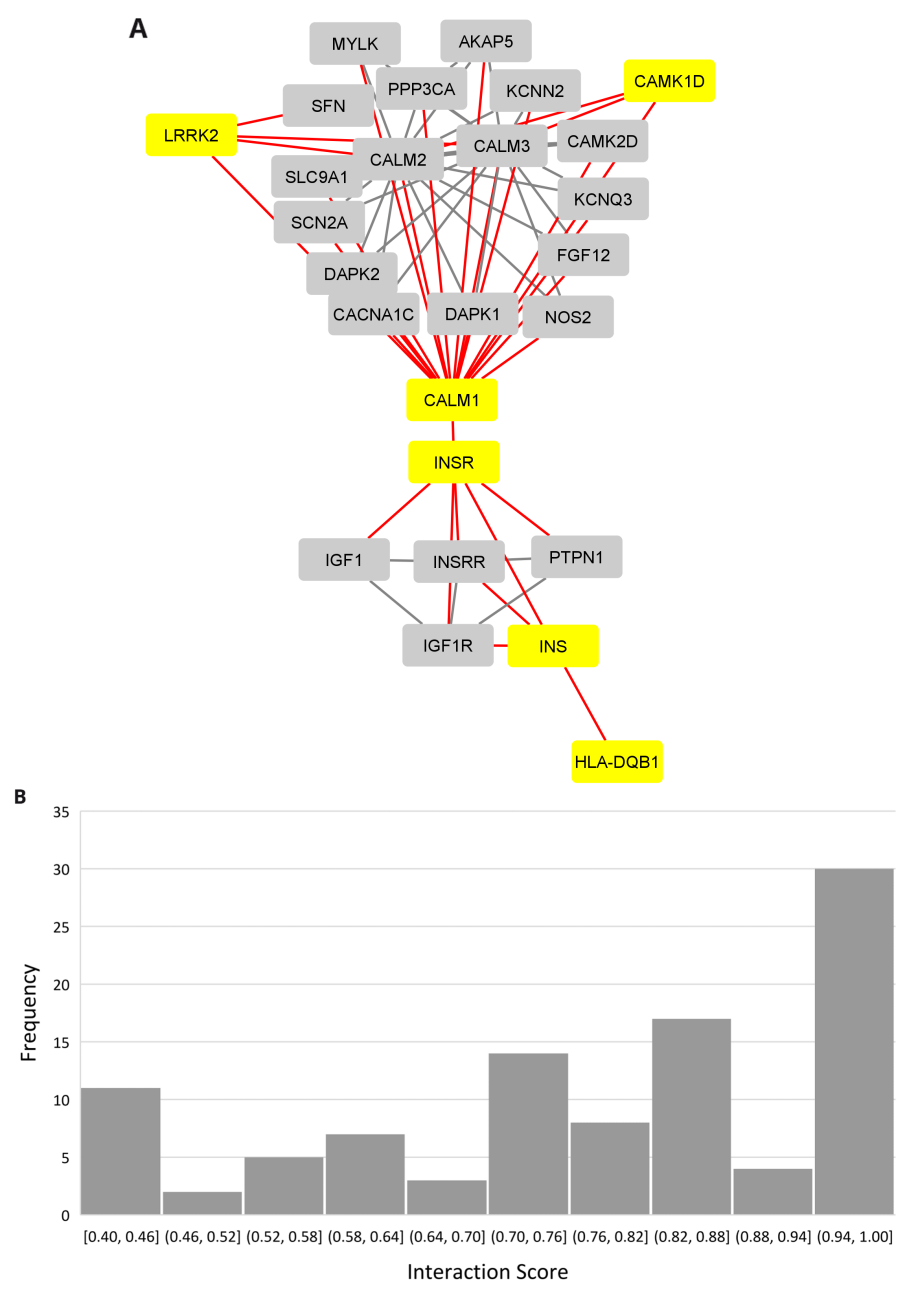
PPI network linking narcolepsy and Parkinson’s through insulin. ( **A**) PPI network showing the insulin interaction with the Narcolepsy gene (HLA-DQB1) and Parkinson's disease gene (LRRK2.) ( **B**) Interaction score distribution, X-axis, interaction score; Y-axis, frequency.

A protein-protein interaction network revealed the insulin connection between the *LRRK2* and *HLA-DQB1* networks using the multiple protein option in the STRING database ( Figure 4A, *Extended data*, Workbook 6, sheets 1–2 ^24^). The distribution for the PPI scores for each show that the majority of the interactions fall in the high range with scores between 0.7 and 1.0 ( Figure 4B). Insulin (INS) and its receptor (INSR) are connected to *HLA-DBQ1* through 1st shell interactions both of which are based on crystallographic evidence. *INSR* is in turn connected to *CALM1*, a calmodulin binding protein involved in calcium signaling and associated with diverse processes including circadian entrainment (KEGG pathway 04713). The evidence for the *INSR/CALM1* interaction is based on coimmunoprecipitation, electro mobility shift, and western blot assays. Relevant interactions, scores, and references are provided in Table 1.

**Table 1.** LRRK2, HLA-DQB1 and CAMK1D relevant network interactions and scores.

PPI	Score	References
INS-INSR	0.974	25
CALM1-CAMK1D	0.732	26
INS-HLA-DQB1	0.72	27
CALM1-INSR	0.433	28
CALM1-LRRK2	0.403	29

In the network, *CALM1* bridges *INSR, CAMK1D* (the only common gene among the database curated genesets for PD, narcolepsy, and IR), and *LRRK2*. The *CALM1* and *CAMK1D* relationship is supported by coimmunoprecipitation and filter binding and phage display assays. The *CALM1/LRRK2* interaction is supported by cosedimentation, coimmunoprecipitation and genetic interference assays.

There are many proteins in the network related to insulin signaling ( *IDE, IGF1, IGF1R, INSRR, YWHAH, YWHAG, PRKCE, KCNN2, INS-IGF2, RAF1, CACNA1C, CALM1, CALM2, CALM3, KCNN2, PRKCE*). Several genes are implicated in AD ( *CACNA1C, CALM1, CALM2, CALM3, IDE*), circadian entrainment ( *CACNA1C, CALM1, CALM2, CALM3*), and dopamine signaling ( *CACNA1C, CALM1, CALM2, CALM3, LRRK2*). *LRRK2* is the only gene in the network linked to PD. There were no experimentally validated interacting partners for *HCRT* and it did not connect to the network.

## Discussion

The aim of this study is to identify the underlying genes and pathways linking PD, narcolepsy, and IR. An integrative genomics and systems biology approach was used for the analysis of gene expression patterns of the *LRRK2, HLA-DQB1,* and *HCRT* genes which are strongly associated with each of these disorders. A comparison of the shared gene correlates for sleep, neurodegeneration, behavior, and insulin led to the identification of genes such as *CACNA1C, BHLHE41, HMGB1,* and *CAMK1D* whose defects might be plausible for the narcoleptic-like symptoms in PD and the relationship with IR.

In addition to the PD- and insulin-related enrichment terms for the *HLA-DQB1* correlates resulting from the cluster analysis, a large number of highly significant genes and terms associated with olfactory processes and keratinocytes/keratin were obtained. The olfaction related genes identified here may be relevant to PD because PD involves degeneration of the olfactory system, which often begins with an impaired sense of smell ^30^. Moreover, diabetes has also been linked to olfactory dysfunction ^31^. There is also evidence supporting a link between PD and keratinocytes and keratin. In a study that considered the effects of PD on non-neuronal tissues, it was reported that individuals with PD had a higher incidence of melanoma and non-melanoma skin cancers ^32^. Interestingly, a link with diabetes and keratinocytes and keratin is most evident in the slow rate of wound healing attributed to high blood glucose levels ^33^.

The PPI network of *LRRK2, HLA-DQB1,* and *CAMK1D* reveals a connection with several insulin/diabetes, circadian, and PD risk factor genes supporting our hypothesis that these three disorders have common pathogenetic processes, further supporting other studies that have reported a relationship between them. Moreover, gene variants of narcolepsy-related genes, *HLA-DQB1* and *CAMK1D*, are associated with diabetes, IR, and PD.

The *RAGE* signaling pathway, identified here through GO enrichment of the shared genesets, may be central to connecting PD and IR. *RAGE* is associated with the pathogenesis of several disorders, including diabetes, via inflammation. *RAGE* is also highly expressed in PD patients when compared with age-matched controls. *RAGE* gene variants have been linked to sporadic PD in an Asian population ^34, 35^, indicating that *RAGE* might play a role in the pathogenesis of PD. Moreover, silencing of the RAGE pathway in a mouse model of PD improved neuroinflammation which causes dopaminergic neurodegeneration in PD patients ^36^. This is important because the deterioration of dopaminergic neurons in the brain is believed to play a critical role in the development of PD ^37^. By the time clinical signs of PD are identified and a diagnosis is made, a large number of dopaminergic neurons are already lost ^1^. Dopaminergic neurons are also involved in promoting feeding behavior in the hypoglycemic state which is mediated by insulin receptors in the substantia nigra, indicating that dopaminergic neuronal loss may alter glycemic control ^38, 39^. Loss of orexin/hypocretin is also linked to binge-eating behavior, low BMR, and obesity, which is also a symptom of narcolepsy. In addition, type 2 diabetes is strongly associated with an increased risk of PD.

Additional insight into the relationship between these disorders is evident from studies in which the repurposing of treatment for one of these diseases has been used to alleviate symptoms in another. Results from a recent clinical trial in which PD patients were treated with intranasal insulin, reported that test subjects had improved verbal fluency and motor skills and sleep related symptoms ^40^. Melatonin, the naturally occurring hormone that controls sleep and wake cycles, was also found to be beneficial in PD ^41^.

There are more than 10 million people worldwide that live with Parkinson's disease. Additional studies aimed at identifying genes and regulatory factors underlying and bridging these comorbid disorders may aid in the design of early intervention and diagnosis strategies, as well as treatment regimes for patients with PD, diabetes, and/or narcolepsy. 

## Conclusion

We have identified the genetic signatures that link PD with its comorbid disorders, narcolepsy and insulin resistance, from the convergence and intersection of dopaminergic, insulin, and immune system related signaling pathways. These findings may aid in the design of early intervention strategies and treatment regimes for non-motor symptoms in PD patients as well as individuals with diabetes and narcolepsy.

## Data availability

### Underlying data

All data underlying the results are available as part of the article and no additional source data are required.

### Extended data

Figshare: Extended data workbook 1 LRRK2, HLA-DQB1, and HCRT gene correlates.xlsx. https://doi.org/10.6084/m9.figshare.13072037.v1 ^17^.

This file contains gene correlates of LRRK2, HLA-DQB1 and HCRT in the hypothalamus, dorsal thalamus, pons and nucleus subcoeruleus.

Figshare: Extended data workbook 2 Cluster analysis of gene correlates.xlsx. https://doi.org/10.6084/m9.figshare.13072103.v1 ^19^.

This file contains cluster analysis of gene correlates of LRRK2, HLA-DQB1 and HCRT in the hypothalamus, dorsal thalamus, pons and nucleus subcoeruleus.

Figshare: Extended data workbook 3 Common genes and functions.xlsx. https://doi.org/10.6084/m9.figshare.13072124.v1 ^20^.

This file contains gene set overlap and functional analysis for LRRK2, HLA-DQB1, and HCRT gene correlates.

Figshare: Extended data workbook 4 Keyword genes.xlsx. https://doi.org/10.6084/m9.figshare.13072130.v1 ^21^.

This file contains keyword enrichment of gene correlates of LRRK2, HLA-DQB1 and HCRT.

Figshare: Extended data workbook 5 PD, narcolepsy and IR risk factors genes.xlsx. https://doi.org/10.6084/m9.figshare.13072151.v1 ^18^.

This file contains Parkinson's disease, narcolepsy and Insulin resistance risk factors genes

Figshare: Extended data workbook 6 LRRK2, HLA-DQB1 and CAMK1D protein-protein interaction network.xlsx. https://doi.org/10.6084/m9.figshare.13072160.v1 ^24^.

This file contains LRRK2, HLA-DQB1 and CAMK1D protein-protein interaction network coordinates.

Extended data are available under the terms of the Creative Commons Attribution 4.0 International license (CC-BY 4.0).
