# Narcolepsy in Parkinson's disease with insulin resistance

**DOI:** 10.12688/f1000research.27413.2

**Published:** 2021-10-20

**Authors:** Alisha Chunduri, Wim E. Crusio, Anna Delprato

**Affiliations:** 1 Department of Biotechnology, Chaitanya Bharathi Institute of Technology, Hyderabad, 500075, India; 2 Department of Research and Education, BioScience Project, Wakefield, MA, 01880, USA; 3 Institut de Neurosciences Cognitives et Intégratives d'Aquitaine, CNRS UMR 5287, Pessac, 33615, France; 4 Institut de Neurosciences Cognitives et Intégratives d'Aquitaine, UMR 5287 University of Bordeaux, Pessac, 33615, France

**Keywords:** Parkinson’s disease, narcolepsy, insulin resistance, diabetes, circadian

## Abstract

**Background: **Parkinson’s disease (PD) is characterized by its progression of motor-related symptoms such as tremors, rigidity, slowness of movement, and difficulty with walking and balance. Comorbid conditions in PD individuals include insulin resistance (IR) and narcolepsy-like sleep patterns. The intersecting sleep symptoms of both conditions include excessive daytime sleepiness, hallucinations, insomnia, and falling into REM sleep more quickly than an average person. Understanding of the biological basis and relationship of these comorbid disorders with PD may help with early detection and intervention strategies to improve quality of life.

**Methods: **In this study, an integrative genomics and systems biology approach was used to analyze gene expression patterns associated with PD, IR, and narcolepsy in order to identify genes and pathways that may shed light on how these disorders are interrelated. A correlation analysis with known genes associated with these disorders *(LRRK2, HLA-DQB1, and HCRT*) was used to query microarray data corresponding to brain regions known to be involved in PD and narcolepsy. This includes the hypothalamus, dorsal thalamus, pons, and subcoeruleus nucleus. Risk factor genes for PD, IR, and narcolepsy were also incorporated into the analysis.

**Results: **The PD and narcolepsy signaling networks are connected through insulin and immune system pathways. Important genes and pathways that link PD, narcolepsy, and IR are *CACNA1C, CAMK1D, BHLHE41, HMGB1,* and AGE-RAGE.

**Conclusions: **We have identified the genetic signatures that link PD with its comorbid disorders, narcolepsy and insulin resistance, from the convergence and intersection of dopaminergic, insulin, and immune system related signaling pathways. These findings may aid in the design of early intervention strategies and treatment regimes for non-motor symptoms in PD patients as well as individuals with diabetes and narcolepsy.

## Introduction

Parkinson’s disease (PD) is characterized by its progression of motor-related symptoms such as tremors, rigidity, slowness of movement, and difficulty with walking and balance ^1– 3^. The motor difficulties associated with PD are attributed to the loss of dopaminergic neurons in the substantia nigra ^1, 4^. There are also non-dopamine lesions that are involved in PD that include the caudal group of intralaminar nuclei (located in dorsal thalamus), and subcoeruleus nuclei ^1, 2^. The main genetic cause of PD is attributed to mutation of the *LRRK2* (leucine-rich repeat kinase 2) gene ^5, 6^.

There is also a significantly increased risk of PD among patients with a history of diabetes and PD associated motor symptoms and cognitive decline are accelerated in individuals with diabetes ^7, 8^). Results from numerous studies investigating the connection between diabetes and PD indicate that there is an overlap in disease mechanisms and pathways particularly in the context of the accumulation of misfolded proteins ^2, 9^, defects in mitochondrial function leading to oxidative stress ^10, 11^, immune system activation resulting in inflammation ^12– 14^, reduced synaptic plasticity, and a decrease in dopamine levels ^15, 16^. Dopamine signaling is also linked to circadian rhythm and sleep ^17^. Studies demonstrate that dopamine levels cycle in a circadian manner in the retina, olfactory bulb, striatum, midbrain, and hypothalamus ^17– 20^. Disrupted sleep patterns and circadian rhythm are also associated with many neuropsychiatric and neurodegenerative illnesses including *LRRK2*-PD and it is often more disturbing than the motor symptoms ^21– 23^.

Most PD patients have daytime sleep attacks and REM sleep disorder that resemble narcolepsy associated sleep symptoms such as excessive daytime drowsiness, sleep paralysis, hallucinations ^24^, and in some cases episodes of cataplexy ^25^. People with narcolepsy frequently enter REM sleep rapidly, within 15 minutes of falling asleep and the muscle weakness or dream activity of REM sleep can occur during wakefulness or be absent during sleep ^26^. Alleles of the *HLA-DQB1* (major histocompatibility complex, class II, DQ beta 1) gene are associated with a predisposition to narcolepsy ^27^, PD ^28^, and Type I diabetes ^29^.

Besides *HLA-DQB1*, the relationship between PD, narcolepsy, and IR may be in part attributed to the hypocretins/orexins which are produced by the *HCRT* gene ^30^. Hypocretins are neurotransmitters that are manufactured by a small number of neurons in the hypothalamus ^31^. They act to stimulate target neurons and promote wakefulness while suppressing rapid-eye-movement (REM) sleep ^32^. Research has shown that there is a massive loss of hypocretin neurons in patients of both PD and narcolepsy and it is hypothesized that the reduction of hypocretin may be the underlying pathogenesis of the narcoleptic symptoms in PD ^24, 32, 33^. In addition to their role in narcolepsy and PD, hypocretins modulate glucose and insulin metabolism ^33^ and also play a critical role in dopamine regulation ^34^. In this study we explore the connection between PD, narcolepsy, and IR using an integrative genomics and systems biology approach.

## Methods

### Genesets and evaluation

Microarray data was collected from the Allen Brain Database using the Human Brain Atlas. To obtain the data, a gene search for *LRRK2, HLA-DQB1*, and *HCRT* was performed. Each of these genes were used to query the atlas for correlates to the hypothalamus, dorsal thalamus, pons, and subcoeruleus nucleus using the dropdown menu for each of the six donor post-mortem brains available in the Allen Human Brain Atlas.

Genes whose expression pattern correlated with *LRRK2, HLA-DQB1*, and *HCRT* were collected for analysis. Correlates with a range of Pearson r values from 0.6 to 1.0 were considered in the analysis ( *Extended data*, Workbook 1 ^35^. The rationale was to investigate genes with a similar expression pattern in order to identify gene correlates specific and common to *LRRK2, HLA-DQB1*, and *HCRT*. Risk factor genes and genes contributing to PD, narcolepsy, and IR were obtained from OMIM, Harmonizome, and GeneWeaver.

Each geneset was evaluated using Gene Ontology (GO) enrichment for clustering, pathways, and keywords using the Database for Annotation, Visualization and Integrated Discovery (DAVID, version 6.8) and the Gene Ontology databases with integrated tools for analysis. Clustering was done in DAVID using the default parameters which include medium stringency settings and a kappa similarity value of 3. The Benjamini corrected P-value was used to determine enrichment significance. The pathway enrichment was performed using KEGG and Panther pathways. The pathways were analyzed manually and evaluated based on shared themes. For the keyword enrichment, a keyword search of the DAVID functional annotation table output was used to identify genes associated with relevant traits related to *LRRK2, HLA-DQB1*, and *HCRT* function. The keywords considered were ‘sleep’, ‘circadian’, ‘parkinson’, ‘locomotion’, ‘dopamine’, ‘behavior’, ‘learning’, ‘memory’, and ‘transcription factor’. Geneset overlap was assessed using Venny 2.0, an online program that compares lists of items to determine the common and unique genes between *LRRK2, HLA-DQB1*, and *HCRT* within and among each brain region (hypothalamus, dorsal thalamus, pons, and subcoeruleus nucleus).

### Network analysis

The String database (version 11.0) was used to build a protein-protein interaction network (ppi for LRRK2, HLA-DQB1, HCRT and CAMK1D which was identified in this study as the only common risk factor gene associated with PD, narcolepsy and IR (Results section: "Functional analysis of PD, narcolepsy and IR risk factor and related genes"). The network was constructed based on experimentally validated interactions using the medium confidence score of 0.4. The combined scores for the interactions are computed by combining the probabilities from the different evidence channels and corrected for the probability of randomly observing an interaction. First and 2nd shell interactions are included in the network. The network was exported from STRING and analyzed in Cytoscape (version 3.7). Network bottlenecks and clusters were identified with Cytoscape plugins CytoHubba (version 0.1) and MCODE (version 1.6.1), respectively. ClueGo (version 2.5.7) was used to analyze the common risk factors and contributing genes for PD, narcolepsy, and IR. The nodes in the network have been manually arranged for proper visibility. Select enriched terms are included in the network ( Figure 3A). All of the enriched terms are provided in *Extended data*, Workbook 5, sheet 5 ^36^. Legacy data sets from the Human Protein Atlas (HPA) ( https://www.proteinatlas.org/) along with gene expression data from the Allen Brain Atlas were used to assess the expression patterns of key genes identified in the ppi network. To assess gene expression of the key network genes in the relevant brain regions considered in this study, we examined microarray data from the Human Allen Brain Atlas for the dorsal thalamus and subcoeruleus nucleus and the HPA for the hypothalamus and pons. Two data sources were used because the Allen Brain Atlas RNA-Seq data did not provide enough samples to evaluate the gene expression in the hypothalamus and pons for these genes. The RNA- Seq data for the dorsal thalamus and subcoeruleus nucleus are derived from either 5 or 6 postmortem donor brains of varying ages and gender. In most instances the genes are sampled multiple times using different probes. The data are expressed as Z score log2 transformed and indicate the number of standard deviations away from the mean. The data across probes was not combined for statistical analysis because it is qualitative but we calculated the percentage of reads with a positive value which measures the detection of RNA at or above the standards used in the assay. This is not to say that the negative values (Number of standard deviations away from the mean) indicate that RNA is not detected.

The HPA includes RNA-Seq data from humans, mouse and pig. Human data are derived from the Genotype-Tissue Expression (GTEx) ( https://www.gtexportal.org/home/) and Functional Annotation of Mammalian Genomes 5 (FANTOM5) projects. Mouse and Pig RNA-Seq data were generated by the Beijing Genomics Institute ( https://www.bgi.com/global/ in collaboration with the HPA). Assay conditions are provided in detail at the HPA ( https://www.proteinatlas.org/about/assays+annotation#transcriptomics).

## Results

### Functional analysis of gene correlates

The cluster analysis for the *LRRK2, HLA-DQB1*, and *HCRT* gene correlates for each brain region resulted in significant enrichment categories for only the *HLA-DQB1* related genesets. For *LRRK2* and *HCRT* there are several instances in which clusters contained enrichment terms for insulin, diabetes, PD, other neurodegenerative disorders, and circadian processes but these did not achieve significance based on the corrected P value criteria. Also, of note for almost every set of correlates, there were many significant enrichment categories and corresponding genes associated with keratinocytes/keratin and olfaction. The clustering results for each set of gene correlates are listed in *Extended data*, Workbook 2 ^37^.

For the *HLA-DQB1* clusters, the significant enrichment terms are: dorsal thalamus: hsa05012:Parkinson's disease (P=4.38E-06), 31 genes and hsa04940:Type I diabetes mellitus (P=0.03), 10 genes; subcoeruleus nucleus: hsa04940:Type I diabetes mellitus (P=6.13E-05), 15 genes, and pons: hsa04940:Type I diabetes mellitus, six genes (P=0.001). The other genes and enrichment categories clustering with PD in the dorsal thalamus are related to mitochondria processes such as oxidative phosphorylation and electron transport as well as Alzheimer’s disease (AD) and Huntington disease (HD).

### Geneset overlap

**Figure 1.**
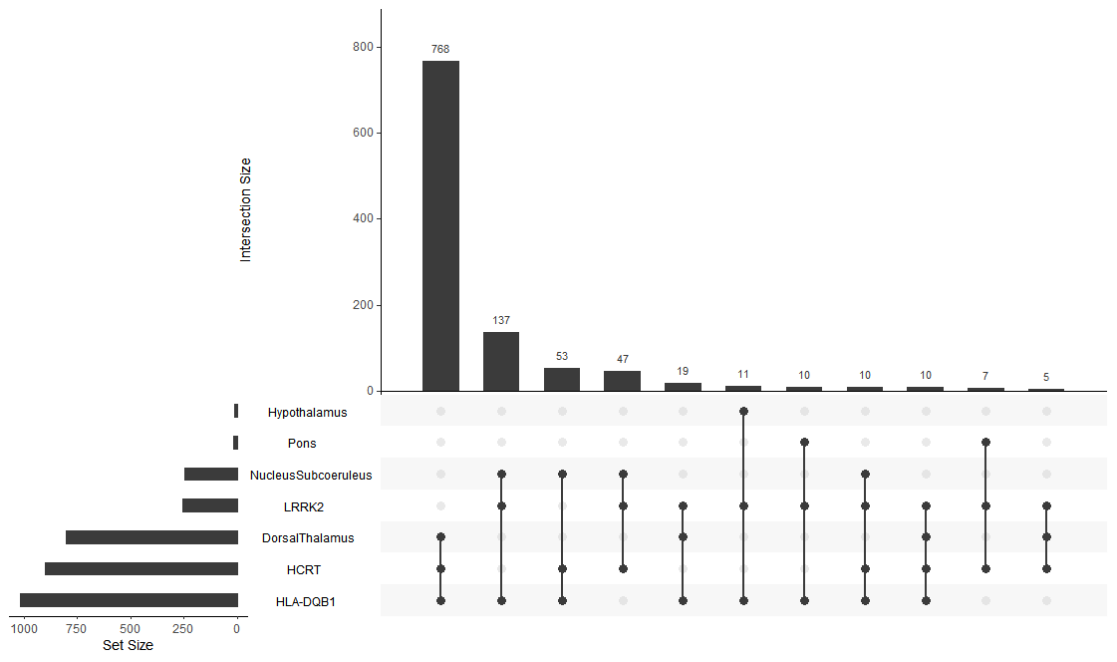
Geneset Overlap. Shared correlates for LRRK2, HLA-DQB1 and HCRT over all brain regions. X-axis, Intersection size; Y-axis, Genes and brain regions.

Among the sets of gene correlates for *LRRK2, HLA-DQB1* and *HCRT*, there are 10 common genes in both the dorsal thalamus and subcoeruleus nucleus ( *A_32_P232747, DISC1, GABRA4, GDF11, HNRNPU, PAK2, , PFKFB2, ROCK1, , SLC9A3R2,* and *ZNF846,* ; Figure 1B). Among the relevant genes are *ROCK1,* which is involved in negative regulation of neuron apoptotic processes, *DISC1* associated with neuron migration, and *HNRNPU* involved in circadian regulation of gene expression. The ten common genes in the subcoeruleus nucleus are *GLYAT, HCN4, HMGB1 HNRNPU, ITGB2,LAMP2, LOC653110, OPA3, ,PHTF2, and SLC6A6, ,* among which the relevant ones to this study are *HNRNPU,* which as mentioned above is involved in circadian regulation of gene expression and insulin signaling, *ITGB2*, which is associated with PD and IR, and *HMGB1,* which is a ligand for the RAGE receptor. Among the sets of gene correlates for *LRRK2, HLA-DQB1*, and *HCRT*, there are no common correlated genes in the hypothalamus and pons.

A detailed description of all shared genes and their associated function for each brain region is provided in *Extended data*, Workbook 3 (sheets 1-8) ^38^. Briefly, the dorsal thalamus and subcoeruleus nucleus have the largest number of shared correlates between *LRRK2, HCRT*, and *HLA-DQB1*. Many of these genes for both brain regions are associated with neuron, insulin, and dopamine related processes. There are also several genes connected directly to PD. In sharp contrast, however, the dorsal thalamus associated correlates have many genes linked to circadian function.

In the dorsal thalamus, the relevant genes are associated with neuron function (negative regulation of neuron apoptotic process, neuron projection development and regulation, neuron differentiation, neuron fate commitment, neuron death in response to oxidative stress, neuron regeneration), circadian processes (regulation of circadian rhythm, circadian entrainment, circadian regulation of gene expression, regulation of circadian rhythm, entrainment of circadian clock by photoperiod), and insulin signaling (insulin secretion, insulin receptor signaling, insulin secretion). Other genes of interest are related to dopamine (dopaminergic neuron differentiation, regulation of dopamine uptake involved in synaptic transmission, positive regulation of dopamine secretion, Wnt signaling pathway involved in midbrain dopaminergic neuron differentiation, dopamine receptor binding, dopamine biosynthetic process, adenylate cyclase-activating dopamine receptor signaling pathway) and also behavior (locomotory, feeding, learning, memory and vocalization, response to stimulants).

In the subcoeruleus nucleus, the relevant genes are also associated with neuron function (negative regulation of neuron differentiation, dopaminergic neuron differentiation, neuron apoptotic process, negative regulation of neuron differentiation, forebrain neuron differentiation), insulin signaling (insulin secretion, insulin receptor signaling pathway, negative regulation of insulin receptor signaling pathway, positive regulation of insulin secretion, diabetes mellitus), dopamine related processes (dopaminergic synapse, dopamine biosynthetic process, dopaminergic neuron differentiation, regulation of synaptic transmission, dopaminergic dopamine biosynthetic process from tyrosine), and behavior (locomotory, vocal learning, response to fear, grooming, response to stimulants).

There are few shared correlated genes in the hypothalamus and pons. For the hypothalamus, the most pertinent genes are involved in neuron migration and circadian processes. In the pons, the relevant genes are concerned with negative regulation of neuron apoptotic processes, neuron projection, circadian regulation of gene expression, and hippocampus and pyramidal neuron development.

Geneset overlap of the correlates for *LRRK2, HLA-DQB1,* and *HCRT* was assessed within each brain region (see *Extended data*, Workbook 3, sheets 9-11) ^38^. Of the *LRRK2* correlates,1.6% were common in all 4 brain regions. Among these are genes associated with insulin ( *MAX, NUCKS1, PIK3R1, PTPN11*), diabetes ( *PIK3R1*) and circadian-related processes ( *HNRNPU, BHLHE41*). Several transcription factors were also present ( *MAX, SKI, ATF7IP, NUCKS1, BHLHE41, NR2C2, PIK3R1*). One of these, *BHLHE41*, acts as a negative regulator of orexin, controls circadian rhythms, and is associated with short sleep syndrome and advanced sleep phase disorder. For *HLA-DQB1*, 1.2% of the correlates are common in all brain regions; relevant associated themes include PD and dopamine ( *SLC18A1*) insulin ( *HLA-DRB5, HLA-DOA, HLA-DQA1, HLA-DQB1*) and transcription factors ( *, FOXE3, HMGB1, LGALS9, PYCARD, SOX8, ZNF446*). Only 0.2% of the *HCRT* correlates are common among all brain regions considered. This includes *HCRT* itself and an insulin associated gene, *GHSR*. Of note, the *MOG* gene, which is present among the *HLA-DQB1* correlates of the subcoeruleus nucleus and *HCRT* correlates of the hypothalamus, is a risk factor for narcolepsy and is linked to PD.

### Keyword evaluation of gene correlates

**Figure 2.**
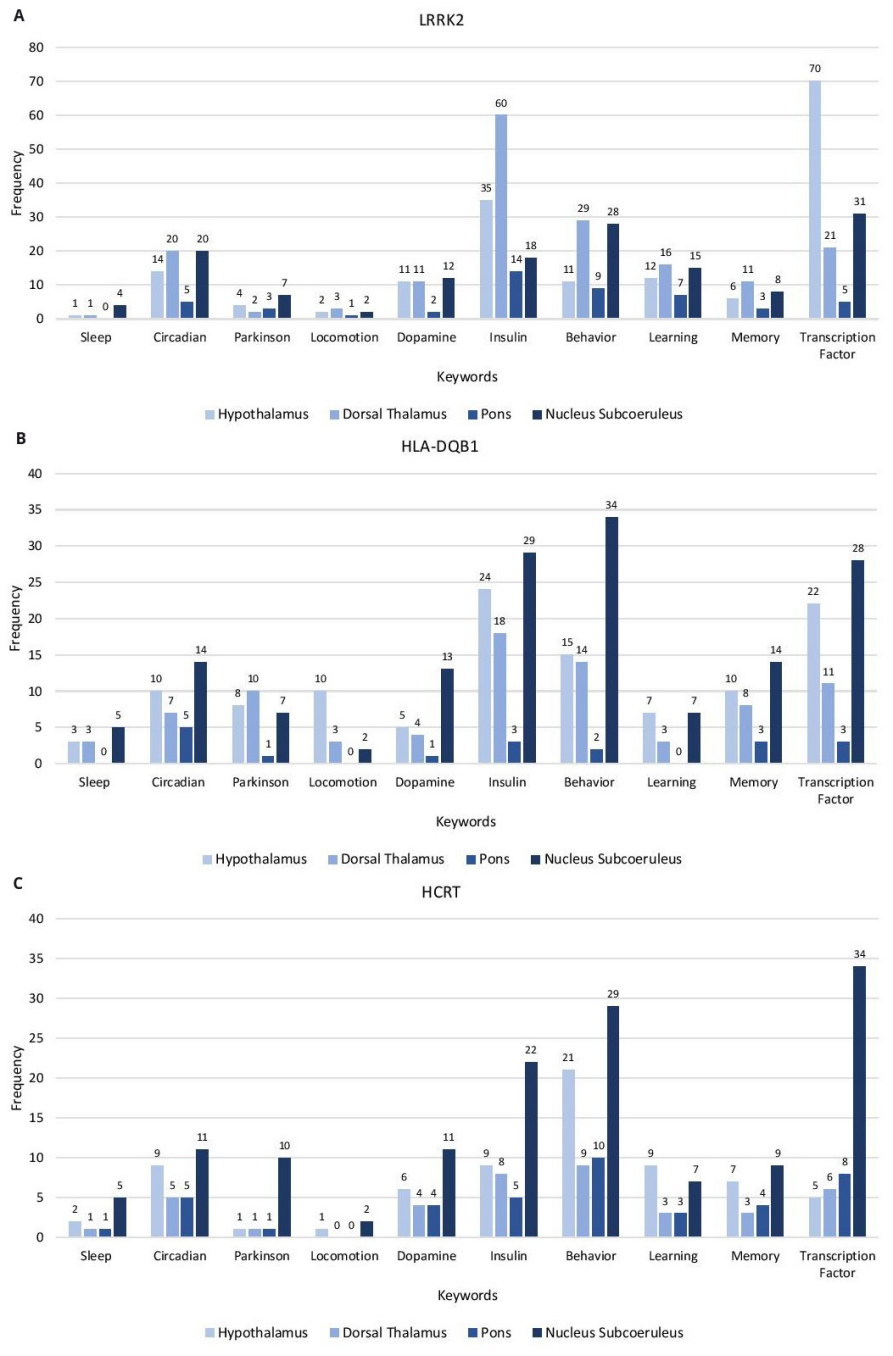
Keyword Enrichment. Representative keyword enrichment of the gene correlates of LRRK2, HLA-DQB1 and HCRT in the Hypothalamus, Dorsal Thalamus, Pons and Nucleus Subcoeruleus based on GO term classification. ( **A**) LRRK2 gene correlates ( **B**) HLA-DQB1 gene correlates ( **C**) HCRT gene correlates. X-axis, keyword categories; Y-axis, frequency of occurrence.

From the GO analysis of the gene correlates, a functional annotation table was generated for GO Biological Process. Genes associated with keywords were obtained and their frequencies determined. The keyword categories used are as follows: sleep, circadian, Parkinson, locomotion, dopamine, insulin, behavior, learning, memory, and transcription factor ( Figure 2A–C and Extended data, Workbook 4, sheets 1-6) ^39^. Each of the correlates for the genesets are evaluated for keywords related to the phenotypes of narcolepsy, PD, and IR in the hypothalamus, dorsal thalamus, pons, and subcoeruleus nucleus. Most of the keywords of the three sets of gene correlates are associated with subcoeruleus nucleus.

The *LRRK2* gene correlates have the highest frequency of the keyword categories. The highest represented categories are: transcription factor (hypothalamus), insulin, behavior, learning, memory, locomotion (dorsal thalamus), dopamine, Parkinson, and sleep (subcoeruleus nucleus) and circadian processes (equal frequency in dorsal thalamus and subcoeruleus nucleus).

The highest represented keyword categories for *HLA-DQB1* are behavior, insulin, transcription factor, circadian, memory, dopamine, sleep (subcoeruleus nucleus), locomotion (hypothalamus), and learning (equal frequencies in the hypothalamus and subcoeruleus nucleus). The highest represented categories of *HCRT* are transcription factor, behavior, insulin, circadian, dopamine, Parkinson, memory, sleep, locomotion (subcoeruleus nucleus), and learning (hypothalamus)

### Functional analysis of PD, narcolepsy, and IR risk factor and related genes

PD, narcolepsy, and IR risk factor and related genesets were evaluated to identify a common set of genes associated with the three disorders ( *Extended data*, Workbook 5, sheets 1-4) ^36^. There were 38 shared genes between the PD and narcolepsy genesets. *CAMK1D* is the only gene common among the 3 genesets for PD, narcolepsy, and IR and it is a Calcium/Calmodulin kinase that is upregulated in PD patients and is also a risk factor for Type 2 diabetes ^40, 41^.

Of the common PD and narcolepsy genes, several were directly associated with PD and narcolepsy behavioral phenotypes such as locomotion ( *DRD2, DRD3, DRD4, GDNF, SLC18A2*), sleep ( *DRD2, DRD3, GRIN2A, HTR2A, NLGN1*), circadian processes ( *CACNA1C, DRD2, DRD3, DRD4, GRIN2, MAPK1, NLGN1, PPARGC1A,*), circadian entrainment ( *CACNA1C, GRIN2A, MAPK1*), learning ( *COMT, DRD1, DRD2, DRD3 GRIN2A*) and memory ( *COMT, DRD1, DRD2, DRD3, GRIN2A, HTR2A*). There were two common genes between the PD and IR genesets: *RREB1,* which is a transcription factor, and *ANKFY1,* which is involved in vesicle trafficking and is also implicated in Type 2 diabetes. There is one common gene between narcolepsy and IR: *HLA-DQB1*, which is the narcolepsy associated gene under study here.

**Figure 3.**
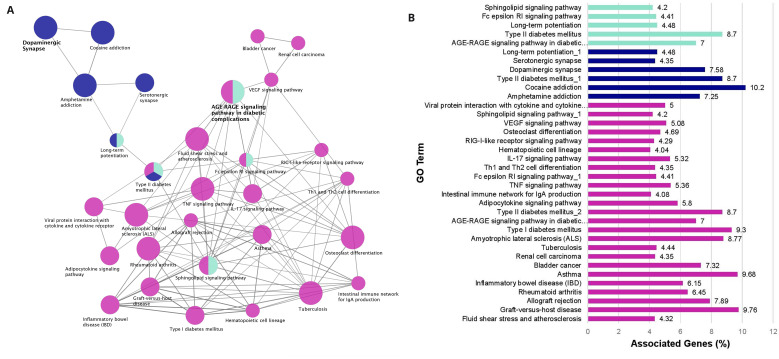
Enrichment Network Analysis. ( **A**) Risk factors enrichment network. In the network the color gradient indicates the proportion of genes in each cluster associated with the enriched GO term. Dark blue nodes include dopaminergic synapse and pathways related to the reward system. Cyan nodes include the AGE-RAGE Signaling pathway in diabetic complications, immune system pathways and lipid signaling. Magenta nodes involve terms associated with immune system function and also insulin signaling. ( **B**) GO pathway terms and associated genes. Bar graph showing the percentage of genes connected with the GO terms. Bars are colored according to the network ( Figure 3A).

The enrichment results are visualized as a network of functionally grouped terms and pathways and listed in the accompanying bar graph ( Figure 3A, B, *Extended data*, Workbook 5, sheet 5) ^36^. The most significant term of a given group is highlighted as the leading term in the network which is indicated by color. The most significant terms emphasized in the graph are dopaminergic synapse (ten genes, KEGG ID:04728, P=2.77E-09) and the AGE-RAGE signaling pathway in diabetic complications (seven genes, KEGG ID:04933, P=4.70E-06) both of which are relevant to PD and IR. Other relevant enriched GO Terms include Type I diabetes mellitus (four genes, KEGG:04940, P=6.27E-04), Type II diabetes mellitus (four genes, KEGG:04930, P=7.54E-04), and Amyotrophic lateral sclerosis (five genes, KEGG:05014, P=9.56E-05). The other enriched terms in the network also represent pathways linked to the reward system, serotonin signaling, immune system function, and insulin regulation. There are several points of convergence in the graph where the enriched terms overlap: AGE-RAGE, Sphingolipid, and Fc Epsilon RI signaling pathways as well as long term potentiation. ( *Extended data*, Workbook 5, sheet 5) ^36^.

### The PD, narcolepsy, and IR connection

**Figure 4.**
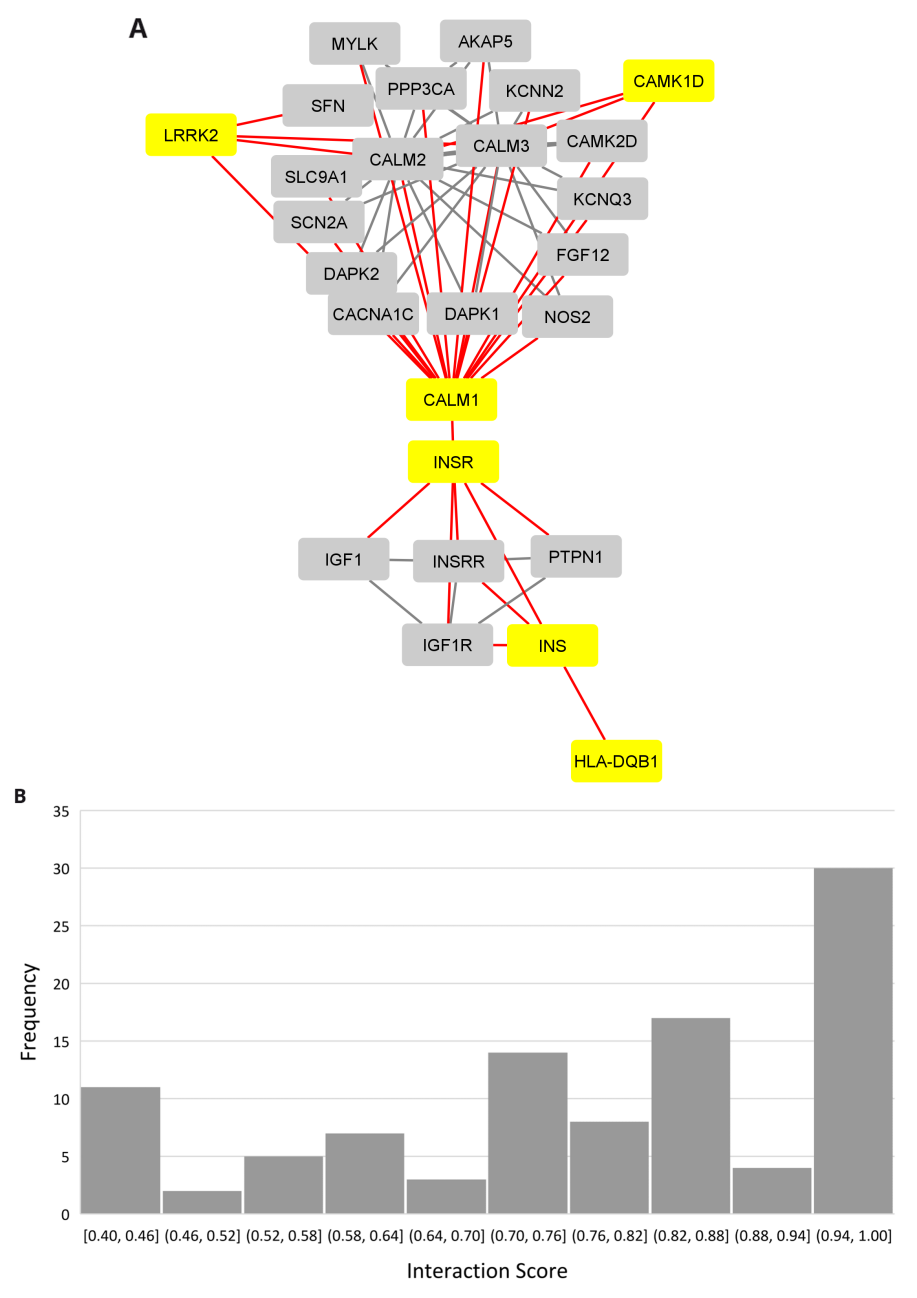
PPI network linking narcolepsy and Parkinson’s through insulin. ( **A**) PPI network showing the insulin interaction with the Narcolepsy gene (HLA-DQB1) and Parkinson's disease gene (LRRK2.) ( **B**) Interaction score distribution,X-axis, interaction score; Y-axis, frequency.

A protein-protein interaction network revealed the insulin connection between the *LRRK2* and *HLA-DQB1* networks using the multiple protein option in the STRING database ( Figure 4A, Extended data, Workbook 6, sheets 1-2) ^42^. The distribution for the PPI scores for each show that the majority of the interactions fall in the high range with scores between 0.7 and 1.0 ( Figure 4B). Insulin (INS) and its receptor (INSR) are connected to *HLA-DBQ1* through 1st shell interactions both of which are based on crystallographic evidence. *INSR* is in turn connected to *CALM1*, a calmodulin binding protein involved in calcium signaling and associated with diverse processes including circadian entrainment (KEGG pathway 04713). The evidence for the *INSR/CALM1* interaction is based on coimmunoprecipitation, electro mobility shift, and western blot assays. Relevant interactions, scores, and references are provided in Table 1.

**Table 1.** LRRK2, HLA-DQB1 and CAMK1D relevant network interactions and scores.

PPI	Score	References
INS-INSR	0.974	55
CALM1-CAMK1D	0.732	56
INS-HLA-DQB1	0.72	57
CALM1-INSR	0.433	58
CALM1-LRRK2	0.403	59

In the network, *CALM1* bridges *INSR, CAMK1D* (the only common gene among the database curated genesets for PD, narcolepsy, and IR), and *LRRK2*. The *CALM1* and *CAMK1D* relationship is supported by coimmunoprecipitation and filter binding and phage display assays. The *CALM1/LRRK2* interaction is supported by cosedimentation, coimmunoprecipitation and genetic interference assays.

There are many proteins in the network related to insulin signaling ( *CACNA1C, CALM1, CALM2, CALM3, IDE, IGF1, IGF1R, INSRR, INS-IGF2, KCNN2, PRKCE, RAF1, YWHAG, YWHAH*). Several genes are implicated in AD ( *CACNA1C, CALM1, CALM2, CALM3, IDE*), circadian entrainment ( *CACNA1C, CALM1, CALM2, CALM3*), and dopamine signaling ( *CACNA1C, CALM1, CALM2, CALM3, LRRK2*). *LRRK2* is the only gene in the network linked to PD. There were no experimentally validated interacting partners for *HCRT* and it did not connect to the network.

Results for gene expression of the key genes in the hypothalamus, and pons, are as follows: Each of the genes with the exception of *AGER* were expressed in both the hypothalamus and pons for human, mouse and pig. *CACNA1C* expression was also confirmed at the protein level by immunocytochemistry in the mouse hypothalamus and pons/medulla Data are provided in *Extended data*, Table 1. The expression pattern of the key genes in dorsal thalamus and subcoeruleus nucleus data were obtained for humans only and are as follows: *HMGB1*, *AGER*, *BHLHE41* are expressed in both the dorsal thalamus and subcoeruleus nucleus. *CACNA1C* is expressed in the dorsal thalamus but is not expressed in the subcoeruleus nucleus. *CAMK1D* was not expressed in either the dorsal thalamus or subcoeruleus nucleus. The results from the gene expression data are summarized in Table 2, Data for the dorsal thalamus and subcoeruleus nucleus are provided in *Extended data*, workbook 7 ^43^.

**Table 2.** Expression summary of key genes in the hypothalamus, dorsal thalamus, nucleus subcoeruleus and pons.

gene	hypothalamus	dorsal thalamus	nucleus subcoeruleus	pons
AGER	-	+	+	-
BHLHE41	+	+	+	+
CACNA1C	+	+	-	+
CAMK1D	+	-	-	+
HMGB1	+	+	+	+

## Discussion

The aim of this study is to identify the underlying genes and pathways linking PD, narcolepsy, and IR. An integrative genomics and systems biology approach was used for the analysis of gene expression patterns of the *LRRK2, HLA-DQB1,* and *HCRT* genes which are strongly associated with each of these disorders. A comparison of the shared gene correlates for sleep, neurodegeneration, behavior, and insulin led to the identification of genes such as *AGER*, *BHLHE41, CACNA1C, CAMK1D*, and *HMGB1*, whose defects might be plausible for the narcoleptic-like symptoms in PD and the relationship with IR.

The ppi network of *LRRK2, HLA-DQB1,* and *CAMK1D* reveals a connection with several insulin/diabetes, circadian, and PD risk factor genes supporting our hypothesis that these three disorders have common pathogenetic processes and further supports earlier studies that have reported a relationship between these conditions. There is a great deal of evidence derived from knockout and cell based studies linking *AGER*, *CACNA1C* and *HMGB1* to Parkinson's pathogenesis. *CACNA1C* is also associated with circadian rhythm and narcolepsy.

There is also evidence of disrupted calcium homeostasis in PD ^44^. Genetic variants of *CACNA1C* which is a subunit of Cav1.2 Ca2+ channels. are linked to greater PD risk which is dependent on vitamin D deficiency ^45^. Microglia in an induced PD model exhibited enhanced neuroinflammation and inhibited neuroprotection in the presence of a Ca2+ agonist ^46^.

Degeneration of dopaminergic neurons have also been observed in microglia-specific Cav1.2 knockdown mice intoxicated with MPTP, a neurotoxin that induces PD-like symptoms ^46^. CACNA1C was also expressed 3x higher in microglia treated with agents to stimulate neuro inflammation ^46^. *CAMK1D*, also associated with Ca2+ signaling, exhibited decreased expression levels in iPSC-derived neurons carrying the *LRRK2* G2019S mutation, the most prevalent genetic cause of late onset PD ^47^.

*HMGB1* and *AGER* appear to act in concert mediating inflammatory processes that ultimately lead to neuron cell death via NF-κB signaling ^48^. Studies indicate that *HMGB*1 is associated with autophagy dysfunction and the degeneration of dopaminergic neurons through interaction with α-synuclein thereby intensifying protein aggregation and in conjunction with RAGE, inflammation and cell death ^49, 50^. RAGE initiates signal transduction cascades and activates NF-κB, increases cytokine expression and also leads to the production of reactive oxygen species ^51, 52^. In neuronal cells expressing the G2019S *LRRK*2 mutant, RAGE enhanced cell death. Expression of RAGE proteins were also upregulated in the LRRK2 mutant cells ^53^. *RAGE* is highly expressed in PD patients when compared with age-matched controls ^51^ and *RAGE* gene variants have been linked to sporadic PD in an Asian population ^54^

Silencing of the RAGE pathway in a mouse model of PD improved neuroinflammation which causes dopaminergic neurodegeneration in PD patients ^52^. This is important because the deterioration of dopaminergic neurons in the brain is believed to play a critical role in the development of PD ^60^. By the time clinical signs of PD are identified and a diagnosis is made, a large number of dopaminergic neurons have already been lost ^1^. Dopaminergic neurons are also involved in promoting feeding behavior in the hypoglycemic state which is mediated by insulin receptors in the substantia nigra, indicating that dopaminergic neuronal loss may alter glycemic control ^61, 62^. Loss of orexin/hypocretin is also linked to binge-eating behavior, low BMR, and obesity, which is a symptom of narcolepsy ^63– 65^.

*BHLHE41*, the other gene of interest identified from the ppi network analysis, is a transcription factor associated with circadian processes. Variants of *BHLHE41* have been implicated in short sleep syndrome ^66^. *BHLHE41* also has a role in immune function and in addition to *CAMK1D*, *AGER* is implicated in diabetes along with the narcolepsy-related gene, *HLA-DQB1.*

 Silencing of the RAGE pathway in a mouse model of PD improved neuroinflammation which causes dopaminergic neurodegeneration in PD patients ^52^. This is important because the deterioration of dopaminergic neurons in the brain is believed to play a critical role in the development of PD ^60^. By the time clinical signs of PD are identified and a diagnosis is made, a large number of dopaminergic neurons are already lost ^1^

Dopaminergic neurons are also involved in promoting feeding behavior in the hypoglycemic state which is mediated by insulin receptors in the substantia nigra, indicating that dopaminergic neuronal loss may alter glycemic control ^61, 62^. Loss of orexin/hypocretin is also linked to binge-eating behavior, low BMR, and obesity, which is also a symptom of narcolepsy ^63– 65^

Further insight into the relationship between PD, IR and disrupted sleep patterns is evident from studies in which the repurposing of treatment for one of these diseases has been used to alleviate symptoms in another. Results from a recent clinical trial in which PD patients were treated with intranasal insulin, reported that test subjects had improved verbal fluency and motor skills and sleep related symptoms ^67^. Insulin is also promising for treating AD symptoms along with growth factors and incretins (orexin) which are a current therapy for T2D ^68^. Metformin another anti diabetic drug has been used to treat AD and may have promise for PD as well ^69^. Current trends in Biomarkers for disease detection include neuroimaging techniques such as petscan monitoring to glucose uptake in PD patients and also monitoring of oxidative stress and cholesterol metabolism ^70, 71^. Melatonin, the naturally occurring hormone that controls sleep and wake cycles, was also found to be beneficial in PD ^72^.

There are more than 10 million people worldwide that live with Parkinson's disease. Additional studies aimed at identifying genes and regulatory factors underlying and bridging these comorbid disorders may aid in the design of early intervention and diagnosis strategies, as well as treatment regimes for patients with PD, diabetes, and/or narcolepsy.

## Conclusion

We have identified genetic signatures that link PD with its comorbid disorders, narcolepsy and insulin resistance, from the convergence and intersection of dopaminergic, insulin, and immune system related signaling pathways. The resulting genes and pathways identified here are consistent with many published findings and may aid in the design of early intervention strategies and treatment regimes for non-motor symptoms in PD patients as well as individuals with diabetes and narcolepsy.

## Data availability

### Source data

All data underlying the results are available as part of the article and no additional source data are required.

### Underlying data

All data underlying the results are available as part of the article and no additional source data are required.

### Extended data

Figshare: Extended data workbook 1 LRRK2, HLA-DQB1, and HCRT gene correlates.xlsx. ( https://doi.org/10.6084/m9.figshare.13072037.v1.

This file contains gene correlates of LRRK2, HLA-DQB1 and HCRT in the hypothalamus, dorsal thalamus, pons and nucleus subcoeruleus.

Figshare: Extended data workbook 2 Cluster analysis of gene correlates.xlsx. https://doi.org/10.6084/m9.figshare.13072103.v1.

This file contains cluster analysis of gene correlates of LRRK2, HLA-DQB1 and HCRT in the hypothalamus, dorsal thalamus, pons and nucleus subcoeruleus.

Figshare: Extended data workbook 3 Common genes and functions.xlsx. https://doi.org/10.6084/m9.figshare.13072124.v1.

This file contains gene set overlap and functional analysis for LRRK2, HLA-DQB1, and HCRT gene correlates.

Figshare: Extended data workbook 4 Keyword genes.xlsx. https://doi.org/10.6084/m9.figshare.13072130.v1.

This file contains keyword enrichment of gene correlates of LRRK2, HLA-DQB1 and HCRT.

Figshare: Extended data workbook 5 PD, narcolepsy and IR risk factors genes.xlsx. https://doi.org/10.6084/m9.figshare.13072151.v1.

This file contains Parkinson's disease, narcolepsy and Insulin resistance risk factors genes

Figshare: Extended data workbook 6 LRRK2, HLA-DQB1 and CAMK1D protein-protein interaction network.xlsx. https://doi.org/10.6084/m9.figshare.13072160.v1.

This file contains LRRK2, HLA-DQB1 and CAMK1D protein-protein interaction network coordinates.

Figshare: Extended data workbook 7 Gene expression patterns for AGER, BHLHE41 CACNA1C, CAMK1D, .HMGB1 in the dorsal thalamus and subcoeruleus nucleus.xlsx. https://doi.org/10.6084/m9.figshare.16680571.

This file contains dorsal thalamus and subcoeruleus nucleus RNA-Seq data for key genes

Figshare: Extended data Table 1. Hypothalamus_and_Pons gene_expression_for_key_genes. https://doi.org 10.6084/m9.figshare.16692085

This file contains hypothalamus and Pons RNA-Seq data for key genes.

Extended data are available under the terms of the Creative Commons Attribution 4.0 International license (CC-BY 4.0).
