# Supplementary material for: Narcolepsy in Parkinson's disease with insulin resistance
Source: F1000Res. 2022 Jan 20;9:1361. Originally published 2020 Nov 23. [Version 3] doi: 10.12688/f1000research.27413.3 (PMC8543173; doi:10.12688/f1000research.27413.3)
Supplement: Peer review history

# Peer review: Reviewer response for version 3

**DOI:** 10.5256/f1000research.119947.r120417

**Published:** 2022-02-03

**Authors:** Makoto Hashimoto

**Affiliations:** 1 Laboratory for Parkinson's disease, Tokyo Metropolitan Institute of Medical Science, Tokyo, Japan

I agree that the manuscript was successfully revised.

Is the work clearly and accurately presented and does it cite the current literature?

Yes

If applicable, is the statistical analysis and its interpretation appropriate?

Yes

Are all the source data underlying the results available to ensure full reproducibility?

Yes

Is the study design appropriate and is the work technically sound?

Yes

Are the conclusions drawn adequately supported by the results?

Partly

Are sufficient details of methods and analysis provided to allow replication by others?

Yes

Reviewer Expertise:

NA

I confirm that I have read this submission and believe that I have an appropriate level of expertise to confirm that it is of an acceptable scientific standard.

# Peer review: Reviewer response for version 3

**DOI:** 10.5256/f1000research.119947.r120416

**Published:** 2022-01-21

**Authors:** Daniel Enterría-Morales

**Affiliations:** 1 Neurobiology Section, Division of Biological Sciences, University of California San Diego, La Jolla, CA, USA

All my comments and questions have been addressed.

Is the work clearly and accurately presented and does it cite the current literature?

Partly

If applicable, is the statistical analysis and its interpretation appropriate?

Yes

Are all the source data underlying the results available to ensure full reproducibility?

Yes

Is the study design appropriate and is the work technically sound?

Partly

Are the conclusions drawn adequately supported by the results?

Partly

Are sufficient details of methods and analysis provided to allow replication by others?

Yes

Reviewer Expertise:

Neurodegeneration, Parkinson's disease, Neurochemistry.

I confirm that I have read this submission and believe that I have an appropriate level of expertise to confirm that it is of an acceptable scientific standard.

# Peer review: Reviewer response for version 2

**DOI:** 10.5256/f1000research.78075.r97492

**Published:** 2021-11-02

**Authors:** Daniel Enterría-Morales

**Affiliations:** 1 Neurobiology Section, Division of Biological Sciences, University of California San Diego, La Jolla, CA, USA

After reviewing the revised version of the manuscript, there are only three minor points that I would like to highlight:

1. This paragraph seems to be duplicated in the "Discussion" section: "Silencing of the RAGE pathway in a mouse model of PD improved neuroinflammation which causes dopaminergic neurodegeneration in PD patients ^52^. This is important because the deterioration of dopaminergic neurons in the brain is believed to play a critical role in the development of PD ^60^. By the time clinical signs of PD are identified and a diagnosis is made, a large number of dopaminergic neurons have already been lost ^_1_^. Dopaminergic neurons are also involved in promoting feeding behavior in the hypoglycemic state which is mediated by insulin receptors in the substantia nigra, indicating that dopaminergic neuronal loss may alter glycemic control ^61,62^. Loss of orexin/hypocretin is also linked to binge-eating behavior, low BMR, and obesity, which is a symptom of narcolepsy ^63–65^".
2. The link to open the Extended data Table 1 has a typographical error. It is listed as https://doi.org 10.6084/m9.figshare.16692085 , but the right link is https://doi.org/10.6084/m9.figshare.16692085 .
3. In the 'Results' section, the authors mention that " *CACNA1C* expression was also confirmed at the protein level by immunocytochemistry in the mouse hypothalamus and pons/medulla. Data are provided in *Extended data*, Table 1". However, in this table there is no data or images showing the immunocytochemistry of CACNA1C in the mouse tissue mentioned above. Also, if this technique has been carried out with mouse tissue, it should be considered as immunohistochemistry. I suggest that the authors add the data to this table, or remove this sentence from this paragraph.

All my comments and questions have been addressed, so the manuscript will be ready for publication after these minor errors have been corrected.

Is the work clearly and accurately presented and does it cite the current literature?

Partly

If applicable, is the statistical analysis and its interpretation appropriate?

Yes

Are all the source data underlying the results available to ensure full reproducibility?

Yes

Is the study design appropriate and is the work technically sound?

Partly

Are the conclusions drawn adequately supported by the results?

Partly

Are sufficient details of methods and analysis provided to allow replication by others?

Yes

Reviewer Expertise:

Neurodegeneration, Parkinson's disease, Neurochemistry.

I confirm that I have read this submission and believe that I have an appropriate level of expertise to confirm that it is of an acceptable scientific standard.

# Response

**Published:** 2022-01-02

**Authors:** Anna Delprato

**Affiliations:** BioScience Project, USA

Thank you for reviewing version 2 of our article and catching these edits. We have incorporated the indicated corrections in a new version.

# Peer review: Reviewer response for version 2

**DOI:** 10.5256/f1000research.78075.r97494

**Published:** 2021-10-21

**Authors:** Qing Wang

**Affiliations:** 1 Department of Neurology, Zhujiang Hospital, Southern Medical University, Guangzhou, Guangdong, China

The authors have fully addressed my concerns, and it would be accepted.

Is the work clearly and accurately presented and does it cite the current literature?

Partly

If applicable, is the statistical analysis and its interpretation appropriate?

Yes

Are all the source data underlying the results available to ensure full reproducibility?

Yes

Is the study design appropriate and is the work technically sound?

Yes

Are the conclusions drawn adequately supported by the results?

Yes

Are sufficient details of methods and analysis provided to allow replication by others?

Yes

Reviewer Expertise:

The authors have fully addressed my concerns, and it would be accepted.

I confirm that I have read this submission and believe that I have an appropriate level of expertise to confirm that it is of an acceptable scientific standard.

# Peer review: Reviewer response for version 1

**DOI:** 10.5256/f1000research.30294.r83029

**Published:** 2021-05-06

**Authors:** Daniel Enterría-Morales

**Affiliations:** 1 Neurobiology Section, Division of Biological Sciences, University of California San Diego, La Jolla, CA, USA

Parkinson’s disease is the second most common neurodegenerative disease right after Alzheimer’s disease nowadays. There is no treatment to counteract the symptoms of the disease, and most of the medications used are aimed at alleviating the symptoms. However, the use of these drugs also has disadvantages in the form of side effects, which makes the quality of life of the patients drop drastically. Lately, research on Parkinson's disease has been focused on the detection of markers that allow diagnosis of this disorder in early stages to increase the probabilities of treatments being effective. Within these types of studies, we have this article published by Chunduri *et al.*, in which the main objective is the search for the expression of certain genes shared between Parkinson's disease, insulin resistance and narcolepsy. The authors propose to analyze the molecular bases of the comorbidity of these three disorders in order to find risk factors that could predispose and facilitate the early diagnosis of Parkinson's disease. As a result of the study, the authors have identified several genes that are related to the signaling pathways of dopamine, insulin and the immune system, among which are: CACNA1C, CAMK1D, BHLHE41, HMGB1, and the AGE-RAGE axis.

 The preliminary data obtained in this study is interesting and would allow further research on the early diagnosis of this disorder, through the individualized study of each of these candidate genes, probably through knock-out or knock-down strategies in animal models. However, some parts of the article need to be supplemented with additional data, and furthermore, the discussion of the data obtained seems short and incomplete, so the authors should increase the number of bibliographic references that are currently discussing the analysis of comorbidity in Parkinson's disease, both in animal models and in humans.

 Specifically, I would like the authors to address the following points:

1. It would be appropriate to validate the expression of some of these candidate genes in the mentioned brain regions, in the tissue of healthy human or wild-type animals (e.g. mouse, rat, monkey), mainly by quantitative PCR or in situ hybridization, or alternatively by Western Blot or immunohistochemistry. It would be very interesting if the authors could also analyze the expression of these genes in tissue from patients or animal models of Parkinson's disease.
2. It would be quite useful to validate the data obtained in humans, with the gene expression database available for mouse or monkey brain tissue, on the Allen Brain Institute website.
  Mouse Brain Atlas: https://developingmouse.brain-map.org/search/index
  Non-human primate Brain Atlas: https://www.blueprintnhpatlas.org/microarray/search
3. It would be great if the authors could comment on why they decided to analyze specifically the comorbidity of Parkinson’s disease with insulin resistance and narcolepsy. There are other disorders that are more frequent comorbidities in Parkinson’s disease patients, such as hypertension, Crohn's disease, schizophrenia, restless leg syndrome, anemia (see attached references). ^1^^,^^2^^,^^3^
4. Could the authors comment and discuss if some of the candidate genes found are expressed in dopaminergic neurons in the human or mouse brain (regarding the bibliography available in the field)?
5. I suggest that the authors should cite more relevant references in the discussion section (see list of references attached). ^4^^,^^5^^,^^6^^,^^7^^,^^8^^,^^9^^,^^10^^,^^11^^,^^12^^,^^13^^,^^14^^,^​​​​​​​ ^15^^,^^16^^,^​​​​​​​ ^17^

Once the authors are able to address all the issues I have proposed in this report, I would be willing to re-evaluate this manuscript.

Is the work clearly and accurately presented and does it cite the current literature?

Partly

If applicable, is the statistical analysis and its interpretation appropriate?

Yes

Are all the source data underlying the results available to ensure full reproducibility?

Yes

Is the study design appropriate and is the work technically sound?

Partly

Are the conclusions drawn adequately supported by the results?

Partly

Are sufficient details of methods and analysis provided to allow replication by others?

Yes

Reviewer Expertise:

Neurodegeneration, Parkinson's disease, Neurochemistry.

I confirm that I have read this submission and believe that I have an appropriate level of expertise to confirm that it is of an acceptable scientific standard, however I have significant reservations, as outlined above.

## References

[1] : Genetic comorbidities in Parkinson's disease. *Hum Mol Genet* .2014;23(3) : 10.1093/hmg/ddt465 831-41 10.1093/hmg/ddt465 24057672PMC3888265

[2] : Comorbid conditions associated with Parkinson's disease: A longitudinal and comparative study with Alzheimer disease and control subjects. *J Neurol Sci* .2017;373: 10.1016/j.jns.2016.12.046 210-215 10.1016/j.jns.2016.12.046 28131190

[3] : Biological and Clinical Implications of Comorbidities in Parkinson's Disease. *Front Aging Neurosci* .2017;9: 10.3389/fnagi.2017.00394 394 10.3389/fnagi.2017.00394 29255414PMC5722846

[4] : DNA variants in CACNA1C modify Parkinson disease risk only when vitamin D level is deficient. *Neurol Genet* .2016;2(3) : 10.1212/NXG.0000000000000072 e72 10.1212/NXG.0000000000000072 27123490PMC4830205

[5] : Blockade of microglial Cav1.2 Ca2+ channel exacerbates the symptoms in a Parkinson’s disease model. *Scientific Reports* .2019;9(1) : 10.1038/s41598-019-45681-3 10.1038/s41598-019-45681-3 31235768PMC6591481

[6] : Parkinson's disease is associated with altered expression of CaV1 channels and calcium-binding proteins. *Brain* .2013;136(Pt 7) : 10.1093/brain/awt134 2077-97 10.1093/brain/awt134 23771339

[7] : High-mobility group box 1 in Parkinson's disease: from pathogenesis to therapeutic approaches. *J Neurochem* .146(3) : 10.1111/jnc.14450 211-218 10.1111/jnc.14450 29676481

[8] : Anti-high mobility group box 1 antibody exerts neuroprotection in a rat model of Parkinson's disease. *Exp Neurol* .2016;275 Pt 1: 10.1016/j.expneurol.2015.11.003 220-31 10.1016/j.expneurol.2015.11.003 26555088

[9] : HMGB1 Mediates Autophagy Dysfunction via Perturbing Beclin1-Vps34 Complex in Dopaminergic Cell Model. *Front Mol Neurosci* .2017;10: 10.3389/fnmol.2017.00013 13 10.3389/fnmol.2017.00013 28197072PMC5281633

[10] : In-vivo evidence that high mobility group box 1 exerts deleterious effects in the 1-methyl-4-phenyl-1,2,3,6-tetrahydropyridine model and Parkinson's disease which can be attenuated by glycyrrhizin. *Neurobiol Dis* .91: 10.1016/j.nbd.2016.02.018 59-68 10.1016/j.nbd.2016.02.018 26921471PMC4867789

[11] : HMGB1 acts on microglia Mac1 to mediate chronic neuroinflammation that drives progressive neurodegeneration. *J Neurosci* .2011;31(3) : 10.1523/JNEUROSCI.3732-10.2011 1081-92 10.1523/JNEUROSCI.3732-10.2011 21248133PMC3046932

[12] : The Receptor for Advanced Glycation Endproducts (RAGE) and Mediation of Inflammatory Neurodegeneration. *J Alzheimers Dis Parkinsonism* .2018;8(1) : 10.4172/2161-0460.1000421 10.4172/2161-0460.1000421 30560011PMC6293973

[13] : RAGE and its emerging role in the pathogenesis of Parkinson’s disease. *Neuroscience Letters* .2018;672: 10.1016/j.neulet.2018.02.049 65-69 10.1016/j.neulet.2018.02.049 29477598

[14] : Receptor for advanced glycation endproducts (RAGE) deficiency protects against MPTP toxicity. *Neurobiol Aging* .2012;33(10) : 10.1016/j.neurobiolaging.2011.12.006 2478-90 10.1016/j.neurobiolaging.2011.12.006 22227007PMC3712169

[15] : AGE-induced neuronal cell death is enhanced in G2019S LRRK2 mutation with increased RAGE expression. *Transl Neurodegener* .2018;7: 10.1186/s40035-018-0106-z 1 10.1186/s40035-018-0106-z 29387348PMC5778750

[16] : The role of advanced glycation end products in various types of neurodegenerative disease: a therapeutic approach. *Cell Mol Biol Lett* .2014;19(3) : 10.2478/s11658-014-0205-5 407-37 10.2478/s11658-014-0205-5 25141979PMC6275793

[17] : Advanced glycation end products (AGEs) and other adducts in aging-related diseases and alcohol-mediated tissue injury. *Exp Mol Med* .2021;53(2) : 10.1038/s12276-021-00561-7 168-188 10.1038/s12276-021-00561-7 33568752PMC8080618

# Response

**Published:** 2021-09-29

**Authors:** Anna Delprato

**Affiliations:** BioScience Project, USA

Reviewer 3

 Parkinson’s disease is the second most common neurodegenerative disease right after Alzheimer’s disease nowadays. There is no treatment to counteract the symptoms of the disease, and most of the medications used are aimed at alleviating the symptoms. However, the use of these drugs also has disadvantages in the form of side effects, which makes the quality of life of the patients drop drastically. Lately, research on Parkinson's disease has been focused on the detection of markers that allow diagnosis of this disorder in early stages to increase the probabilities of treatments being effective. Within these types of studies, we have this article published by Chunduri et al., in which the main objective is the search for the expression of certain genes shared between Parkinson's disease, insulin resistance and narcolepsy. The authors propose to analyze the molecular bases of the comorbidity of these three disorders in order to find risk factors that could predispose and facilitate the early diagnosis of Parkinson's disease. As a result of the study, the authors have identified several genes that are related to the signaling pathways of dopamine, insulin and the immune system, among which are: CACNA1C, CAMK1D, BHLHE41, HMGB1, and the AGE-RAGE axis.

 The preliminary data obtained in this study is interesting and would allow further research on the early diagnosis of this disorder, through the individualized study of each of these candidate genes, probably through knock-out or knock-down strategies in animal models.

 However, some parts of the article need to be supplemented with additional data, and furthermore, the discussion of the data obtained seems short and incomplete, so the authors should increase the number of bibliographic references that are currently discussing the analysis of comorbidity in Parkinson's disease, both in animal models and in humans.

 Reviewer 3 comment:

 Specifically, I would like the authors to address the following points:

 It would be appropriate to validate the expression of some of these candidate genes in the mentioned brain regions, in the tissue of healthy human or wild-type animals (e.g. mouse, rat, monkey), mainly by quantitative PCR or in situ hybridization, or alternatively by Western Blot or immunohistochemistry. It would be very interesting if the authors could also analyze the expression of these genes in tissue from patients or animal models of Parkinson's disease.

**Response:**

**We have addressed the request for validation of candidate gene expression in the mentioned brain regions above in our response to Reviewer 1 and have added the information to the Results and Discussion sections of the revised manuscript.** **To summarize our findings, each of the key genes is expressed in at least two of the brain regions relevant to this study.**

**To address the reviewer’s question concerning expression of these genes in tissue from patients or animal models of Parkinson's disease, we have analyzed the gene expression data available in the NCBI GEO database specifically for Parkinson’s Disease datasets to determine if any of the key genes identified in our study are differentially expressed.**

**Out of 24 available datasets, we found that BHLHE41 and AGER were differentially expressed as compared with controls from two distinct studies ( https://www.ncbi.nlm.nih.gov/geo/geo2r/?acc=GSE36321 and https://www.ncbi.nlm.nih.gov/geo/query/acc.cgi?acc=GSE19587).**

**For BHLHE41, the study involved assessing gene expression in human neural stem cells containing the LRRK2 (G2019S) pathogenic mutation. The LRRK2 (G2019S) containing cells had higher expression of BHLHE4. AGER was differentially expressed with borderline significance in a study that examined post mortem medullary regions from brains with evidence of Parkinson’s. AGER was expressed higher in brains from individuals with Parkinson’s. However, due to low n values we do not have enough confidence in the data to include these results in the manuscript.** **The other genes of interest, CACNA1C, CAMK1D, and HMGB1 were not identified in any of the GEO gene expression datasets as being expressed differently than controls.**

**We also assessed expression of the key genes in individuals with Parkinson’s versus a healthy control group from data generated in a longitudinal study in which the aim is to identify biomarkers of Parkinson’s disease progression (Parkinson's Progression Markers Initiative** **https://www.ppmi-info.org/). The Parkinson’s cohort consisted of 423 individuals and the control group, 196. There was no difference in the expression for any of the key genes.**

 Reviewer 3 comment:

 It would be quite useful to validate the data obtained in humans, with the gene expression database available for mouse or monkey brain tissue, on the Allen Brain Institute website.

 Mouse Brain Atlas: https://developingmouse.brain-map.org/search/index

 Non-human primate Brain Atlas: https://www.blueprintnhpatlas.org/microarray/search

**Response:**

**Thank you for the suggestion. However, to the best of our knowledge, there is no Microarray data available for mouse at the Allen Brain Atlas but we were able to consider the in situ hybridization data (discussed above). Regrettably the Non-human primate atlas does not contain data for the relevant brain regions used in this study which is unfortunate because this is an intriguing dataset.**

 Reviewer 3 comment: 

 It would be great if the authors could comment on why they decided to analyze specifically the comorbidity of Parkinson’s disease with insulin resistance and narcolepsy. There are other disorders that are more frequent comorbidities in Parkinson’s disease patients, such as hypertension, Crohn's disease, schizophrenia, restless leg syndrome, anemia (see attached references).1,2,3

**Response:**

**Initially, the focus of our study was to investigate the connection between Parkinson’s and narcolepsy. Early on in the data collection and preliminary analysis, we observed that many of the gene correlates were linked to insulin resistance. On the basis of this finding we decided to expand the scope of the study.**

 Reviewer 3 comment: 

 Could the authors comment and discuss if some of the candidate genes found are expressed in dopaminergic neurons in the human or mouse brain (regarding the bibliography available in the field)?

**Response:**

**AGER, BHLHE41, CACNA1C, and HMGB1 are expressed in Dopaminergic neurons from the substantia nigra pars compacta and ventral tegmental area in rats. These data are publicly accessible at GEO datasets, accession number: GSE1837 ( https://www.ncbi.nlm.nih.gov/geo/query/acc.cgi?acc=GSE1837** **).**

**There is also further support from previously published studies:**

**CACNA1C (PTSD study generated mice with specific deletion of cacna1c from D1R-expressing neurons https://pubmed.ncbi.nlm.nih.gov/32332995/).**

**CAMK1D (Decreased mRNA Expression of Key ER Ca2+ Regulators and CamK1D in LRRK2 G2019S NeuronsD https://www.sciencedirect.com/science/article/pii/S2213671118304909)**

**HMGB1/RAGE/AGER (Activation of the HMGB1-RAGE axis upregulates TH expression in dopaminergic neurons via JNK phosphorylation https://pubmed.ncbi.nlm.nih.gov/28887039)**

 Reviewer 3 comment:

 I suggest that the authors should cite more relevant references in the discussion section (see list of references attached).4,5,6,7,8,9,10,11,12,13,14,15,16,17

**Response:**

**We thank the reviewer for providing these references. We have updated the Introduction and Discussion sections of the revised manuscript to include this information.**

# Peer review: Reviewer response for version 1

**DOI:** 10.5256/f1000research.30294.r82398

**Published:** 2021-04-12

**Authors:** Qing Wang

**Affiliations:** 1 Department of Neurology, Zhujiang Hospital, Southern Medical University, Guangzhou, Guangdong, China

This study has identified the genes such as *CACNA1C, CAMK1D,* *BHLHE41, HMGB1, *and AGE-RAGE that link PD with narcolepsy and insulin resistance and their signaling networks are connected through insulin and immune system pathways. These findings may contribute to develop new treatment strategies in PD patients as well as individuals with diabetes and narcolepsy. The results of the study are interesting.

 However, the introduction and discussion are too weak. So it is suggested to cite more relevant references as follows (please see the reference list below) in order to elaborate on the mechanisms.  

 After my concerns above have been fully addressed, I am happy to re-evaluate this manuscript.

Is the work clearly and accurately presented and does it cite the current literature?

Partly

If applicable, is the statistical analysis and its interpretation appropriate?

Yes

Are all the source data underlying the results available to ensure full reproducibility?

Yes

Is the study design appropriate and is the work technically sound?

Yes

Are the conclusions drawn adequately supported by the results?

Yes

Are sufficient details of methods and analysis provided to allow replication by others?

Yes

Reviewer Expertise:

NA

I confirm that I have read this submission and believe that I have an appropriate level of expertise to confirm that it is of an acceptable scientific standard, however I have significant reservations, as outlined above.

## References

[1] : Antidiabetic Drug Metformin Ameliorates Depressive-Like Behavior in Mice with Chronic Restraint Stress via Activation of AMP-Activated Protein Kinase. *Aging Dis* .2020;11(1) : 10.14336/AD.2019.0403 31-43 10.14336/AD.2019.0403 32010479PMC6961762

[2] : Vascular, inflammatory and metabolic risk factors in relation to dementia in Parkinson’s disease patients with type 2 diabetes mellitus. *Aging* .2020;12(15) : 10.18632/aging.103776 15682-15704 10.18632/aging.103776 PMC746739032805719

[3] : The Metabolic Activity of Caudate and Prefrontal Cortex Negatively Correlates with the Severity of Idiopathic Parkinson's Disease. *Aging Dis* .2019;10(4) : 10.14336/AD.2018.0814 847-853 10.14336/AD.2018.0814 31440389PMC6675526

[4] : Ketogenic Diet Ameliorates Cardiac Dysfunction via Balancing Mitochondrial Dynamics and Inhibiting Apoptosis in Type 2 Diabetic Mice. *Aging Dis* .2020;11(2) : 10.14336/AD.2019.0510 229-240 10.14336/AD.2019.0510 32257538PMC7069456

[5] : "Hot cross bun" is a potential imaging marker for the severity of cerebellar ataxia in MSA-C. *NPJ Parkinsons Dis* .2021;7(1) : 10.1038/s41531-021-00159-w 15 10.1038/s41531-021-00159-w 33589630PMC7884406

[6] : BDNF Alleviates Neuroinflammation in the Hippocampus of Type 1 Diabetic Mice via Blocking the Aberrant HMGB1/RAGE/NF-κB Pathway. *Aging Dis* .2019;10(3) : 10.14336/AD.2018.0707 611-625 10.14336/AD.2018.0707 31165005PMC6538223

[7] : Disease Progression-Dependent Expression of CD200R1 and CX3CR1 in Mouse Models of Parkinson's Disease. *Aging Dis* .2020;11(2) : 10.14336/AD.2019.0615 254-268 10.14336/AD.2019.0615 32257540PMC7069458

[8] : Various Diseases and Clinical Heterogeneity Are Associated With "Hot Cross Bun". *Front Aging Neurosci* .2020;12: 10.3389/fnagi.2020.592212 592212 10.3389/fnagi.2020.592212 33328971PMC7714952

[9] : Multimodal analysis of gene expression from postmortem brains and blood identifies synaptic vesicle trafficking genes to be associated with Parkinson’s disease. *Briefings in Bioinformatics* .2020; 10.1093/bib/bbaa244 10.1093/bib/bbaa244 33079984

[10] : Salsalate Prevents β-Cell Dedifferentiation in OLETF Rats with Type 2 Diabetes through Notch1 Pathway. *Aging Dis* .2019;10(4) : 10.14336/AD.2018.1221 719-730 10.14336/AD.2018.1221 31440379PMC6675521

[11] : Cystatin C as a potential therapeutic mediator against Parkinson’s disease via VEGF-induced angiogenesis and enhanced neuronal autophagy in neurovascular units. *Cell Death & Disease* .2017;8(6) : 10.1038/cddis.2017.240 10.1038/cddis.2017.240 PMC552089928569795

[12] : Neurological Manifestation of Incretin-Based Therapies in Patients with Type 2 Diabetes: A Systematic Review and Network Meta-Analysis. *Aging Dis* .2019;10(6) : 10.14336/AD.2019.0303 1311-1319 10.14336/AD.2019.0303 31788342PMC6844583

[13] : Rapid Eye Movement Sleep Behavior Disorder and Neurodegenerative Diseases: An Update. *Aging Dis* .2020;11(2) : 10.14336/AD.2019.0324 315-326 10.14336/AD.2019.0324 32257544PMC7069464

[14] : Potential Biochemical Mechanisms of Brain Injury in Diabetes Mellitus. *Aging Dis* .2020;11(4) : 10.14336/AD.2019.0910 978-987 10.14336/AD.2019.0910 32765958PMC7390528

[15] : Lee Silverman Voice Treatment for dysarthria in patients with Parkinson's disease: a systematic review and meta-analysis. *Eur J Neurol* .27(10) : 10.1111/ene.14399 1957-1970 10.1111/ene.14399 32539227

[16] : Contra-Directional Expression of Plasma Superoxide Dismutase with Lipoprotein Cholesterol and High-Sensitivity C-reactive Protein as Important Markers of Parkinson's Disease Severity. *Front Aging Neurosci* .2020;12: 10.3389/fnagi.2020.00053 53 10.3389/fnagi.2020.00053 32210787PMC7068795

# Response

**Published:** 2021-09-29

**Authors:** Anna Delprato

**Affiliations:** BioScience Project, USA

Reviewer 2 

 This study has identified the genes such as CACNA1C, CAMK1D, BHLHE41, HMGB1, and AGE-RAGE that link PD with narcolepsy and insulin resistance and their signaling networks are connected through insulin and immune system pathways. These findings may contribute to developing new treatment strategies in PD patients as well as individuals with diabetes and narcolepsy. The results of the study are interesting.

 Reviewer 2 comment:

 However, the introduction and discussion are too weak. So it is suggested to cite more relevant references as follows (please see the reference list below) in order to elaborate on the mechanisms. After my concerns above have been fully addressed, I am happy to re-evaluate this manuscript.

**Response:**

**We thank the reviewer for providing us with many relevant references. Based on this information we have revised the Introduction and Discussion sections of the manuscript and have included more mechanistic detail and context as recommended.**

# Peer review: Reviewer response for version 1

**DOI:** 10.5256/f1000research.30294.r77250

**Published:** 2021-02-08

**Authors:** Makoto Hashimoto

**Affiliations:** 1 Laboratory for Parkinson's disease, Tokyo Metropolitan Institute of Medical Science, Tokyo, Japan

Comorbidity of Parkinson’s disease (PD) with insulin resistance (IR) and narcolepsy-like sleep patterns are frequently described, but the mechanism is unclear.  

 In this study, an integrative genomics and systems biology approach was used to analyze gene expression patterns associated with PD, IR, and narcolepsy to identify genes and pathways that may shed light on how these disorders are interrelated. The results showed that the PD and narcolepsy signaling networks are connected through insulin and immune system pathways. Important genes and pathways that link PD, narcolepsy, and IR were shown to be *CACNA1C, CAMK1D, BHLHE41, HMGB1,* and AGE-RAGE. The authors concluded that these findings might aid in the design of early intervention strategies and treatment regimes for non-motor symptoms in PD patients as well as individuals with diabetes and narcolepsy.

 Overall, the results are interesting and clear. I agree that the paper may contribute to the PD research. My comments are as follows:

1. It would be much better if modification of the expression of the identified genes (knockout or overexpression), including *CACNA1C, CAMK1D, BHLHE41, HMGB1,* and AGE-RAGE, might result in phenotypes related to PD and/or narcolepsy in cells or animal models. Are there such previous papers? Otherwise, the result of network analysis alone seems preliminary.
2. In the same context, other experiments, such as immunohistochemistry, should be conducted to confirm the expression of these genes at the protein level.

Is the work clearly and accurately presented and does it cite the current literature?

Yes

If applicable, is the statistical analysis and its interpretation appropriate?

Yes

Are all the source data underlying the results available to ensure full reproducibility?

Yes

Is the study design appropriate and is the work technically sound?

Yes

Are the conclusions drawn adequately supported by the results?

Partly

Are sufficient details of methods and analysis provided to allow replication by others?

Yes

Reviewer Expertise:

neurodegenerative diseases

I confirm that I have read this submission and believe that I have an appropriate level of expertise to confirm that it is of an acceptable scientific standard, however I have significant reservations, as outlined above.

# Response

**Published:** 2021-03-10

**Authors:** Anna Delprato

**Affiliations:** BioScience Project, USA

We would like to thank you for taking the time to review our article and providing constructive feedback. We will submit a revised version once we have received all of the reviews.

# Response

**Published:** 2021-09-29

**Authors:** Anna Delprato

**Affiliations:** BioScience Project, USA

Reviewer 1

 Comorbidity of Parkinson’s disease (PD) with insulin resistance (IR) and narcolepsy-like sleep patterns are frequently described, but the mechanism is unclear. 

 In this study, an integrative genomics and systems biology approach was used to analyze gene expression patterns associated with PD, IR, and narcolepsy to identify genes and pathways that may shed light on how these disorders are interrelated. The results showed that the PD and narcolepsy signaling networks are connected through insulin and immune system pathways. Important genes and pathways that link PD, narcolepsy, and IR were shown to be CACNA1C, CAMK1D, BHLHE41, HMGB1, and AGE-RAGE. The authors concluded that these findings might aid in the design of early intervention strategies and treatment regimes for non-motor symptoms in PD patients as well as individuals with diabetes and narcolepsy.

 Overall, the results are interesting and clear. I agree that the paper may contribute to the PD research. My comments are as follows:

 Reviewer 1 comment:

 It would be much better if modification of the expression of the identified genes (knockout or overexpression), including CACNA1C, CAMK1D, BHLHE41, HMGB1, and AGE-RAGE, might result in phenotypes related to PD and/or narcolepsy in cells or animal models. 

 Are there such previous papers? Otherwise, the result of network analysis alone seems 

 Preliminary.

**Response:**

**We thank the reviewer for taking the time to provide us constructive feedback on this manuscript. There is in fact a great deal of evidence derived from knockout and cell based studies linking CACNA1C, HMGB1, and AGER/AGE-RAGE to Parkinson's pathogenesis. CACNA1C is also associated with Circadian rhythm and narcolepsy. In addition, CAMK1D is a component of the calmodulin dependent calcium signaling cascade and there is also support for its role as an interactor of LRRK2, one of the key genes influencing Parkinson’s. HMGB1 and AGER/AGE-RAGE appear to act in concert mediating inflammatory processes that ultimately lead to neuron cell death via NF-κB signaling. AGER/AGE-RAGE and LRRK2 are also linked as RAGE proteins are upregulated in LRRK2 G2019S mutant cells. The LRRK2 G2019S mutation is the most common genetic cause of neurodegeneration and PD. The other major gene of interest identified in the ppi network, BHLHE41, is a transcription factor associated with Circadian processes. Variants of BHLHE41 have been implicated in short sleep syndrome and this gene also has a role in immune function. BHLHE41 in addition to CAMK1D, RAGE, and HMGB1 are also implicated in diabetes.**

**Details of the supporting studies for the candidate genes as well as the other network genes have been included in the Introduction and Discussion sections of the revised manuscript.**

 Reviewer 1 comment:

 In the same context, other experiments, such as immunohistochemistry, should be conducted to confirm the expression of these genes at the protein level.

**Response:**

**We agree that the above mentioned studies could be useful to further support the results presented here. Regrettably we do not have access to wet lab facilities. Our method of research is based in bioinformatics, systems biology and integrative genomics.**

**However, we were able to address your question using legacy datasets from the Human Protein Atlas ( https://www.proteinatlas.org/) which also contains data for gene expression in mice and pigs and the human database in the Allen Brain Atlas ( https://human.brain-map.org/microarray/search).**

**Results for gene expression of the key genes in the hypothalamus and pons are as follows: Each of the genes with the exception of AGER were expressed in both the hypothalamus and pons for human, mouse and pig. CACNA1C expression was also confirmed at the protein level by immunocytochemistry in the mouse hypothalamus and pons/medulla.**

**The expression pattern of the key genes in dorsal thalamus and subcoeruleus nucleus data were obtained for humans only and are as follows: HMGB1, AGER, BHLHE41 are expressed in both the dorsal thalamus and subcoeruleus nucleus. CACNA1C is expressed in the dorsal thalamus and is not expressed in the subcoeruleus nucleus CAMK1D was not expressed in either the dorsal thalamus or subcoeruleus nucleus.** **These data are have been added to the revised version of the manuscript.**
